# Measuring COVID-19 Related Anxiety in Parents: Psychometric Comparison of Four Different Inventories

**DOI:** 10.2196/24507

**Published:** 2020-12-03

**Authors:** Christian Kubb, Heather M Foran

**Affiliations:** 1 Health Psychology Unit Institute of Psychology Universität Klagenfurt Klagenfurt Austria

**Keywords:** COVID-19, coronavirus, anxiety, parents, parenting, scale, inventory, well-being, mental health, stress

## Abstract

**Background:**

The COVID-19 outbreak and the measures to contain the global pandemic can have an impact on the well-being and mental health status of individuals. Parents of young children are particularly at risk for high levels of parental stress due to the current public health crisis, which can impact parenting behaviors and children’s well-being. Although different initial scales have been developed to measure COVID-19–related anxiety, they have not yet been tested sufficiently in parent samples. A brief measure of COVID-19–related anxiety is necessary for both quick assessment in practice and in larger epidemiological studies of parents.

**Objective:**

The purpose of this study is to compare the distributions, validities, and reliabilities of four different COVID-19 anxiety scales: Fear of COVID-19 Scale, Coronavirus Anxiety Scale, Pandemic Anxiety Scale, and one subscale of the COVID Stress Scales. Based on the psychometric properties of these scales, we aim to provide recommendations for a brief unidimensional inventory to assess COVID-19–related anxiety among parents.

**Methods:**

A cross-sectional web-based survey of 515 German-speaking parents (465 mothers, 90.3%) with at least one child aged 0-6 years was conducted during a 6-week period (June 29 to August 9, 2020). Half of the parents were recruited via Facebook parenting groups, while the other half were recruited through childcare centers. We psychometrically tested 25 items on COVID-19–related anxiety using the framework of classical test theory, including item analysis, correlational analysis of family variables, and exploratory factor analysis. Moreover, an item response theory approach was applied to estimate item discriminations and item difficulties.

**Results:**

Based on the psychometric properties, three items of the Pandemic Anxiety Scale were identified as a single unidimensional factor. The adapted scale demonstrated acceptable internal consistency (α=.79), moderate to high item discrimination, strong positive intercorrelation with two other COVID-19 anxiety scales, and a small positive association with parenting stress. Mothers and fathers did not differ in total scores (t_513_=−0.79, *P*=.42).

**Conclusions:**

Factor analysis suggests that existing COVID-19–related anxiety scales measure different latent constructs of anxiety. Furthermore, all scales showed only small to moderate correlations with trait health anxiety, suggesting that COVID-19–related anxiety is distinct from general health anxiety. The adapted “disease anxiety” subscale of the Pandemic Anxiety Scale is an economical measure for assessing COVID-19–related anxiety in parents. Directions for future research are outlined.

## Introduction

### Background

In December 2019, patients with unusual cases of pneumonia in Wuhan City, China, were reported [1]; later, in January 2020, this pneumonia was identified as being caused by the pathogen SARS-CoV-2 [2]. Confirmed individual cases and clusters were subsequently observed in almost all countries worldwide [3,4]. On March 11, 2020, the World Health Organization [5] declared the rapidly spreading COVID-19 to be a global pandemic. Shortly afterward, many countries worldwide went into lockdown, with measures of closed borders, social distancing, and quarantine orders, to curb the spread of the novel coronavirus. To date (October 14, 2020), over 38 million confirmed cases of COVID-19 and 1,080,000 deaths have been reported by Johns Hopkins University [4].

Despite the large discrepancies in infection and death rates within Europe, the socioeconomic impact of the adopted measures for disease control has been more or less equal in all countries. Since then, notable effects on physical and mental health have been widely described, including symptoms of anxiety, stress, and depression [6-8]. The danger of a global mental health crisis was discussed early in the pandemic due to manifold restrictions and changes in daily living: confrontation with death, fear of contracting the virus or transmitting it to others, isolation during quarantine, financial strain, job loss, anger against measures, closed public facilities, and visitation bans to relatives [9,10]. These severe life events can exacerbate existing mental disorders and increase the risk of new incidences of stress-related disorders [11,12].

In particular, the pandemic outbreak has confronted families with unprecedented and immediate challenges in their daily routines. Psychosocial and economic changes have challenged family life. In the United States alone, the number of employed people decreased by 20 million between February and May 2020 [13]. Increased levels of stress and anxiety were found in parents [14-19], with substantial strains in compatibility with intensive child care responsibilities and employment while facilities were closed [7]. As a result, additional problems such as homeschooling and crowded households have emerged and placed additional strain on parents [7,20,21]. During the COVID-19 crisis, parents are may at increased risk for suffering from parental burnout [22], with growing evidence that mothers, children, and immigrants from households with low socioeconomic status are most affected by negative mental and physical health sequelae [23-27].

Perceived danger and fear of health consequences can be expected during pandemic outbreaks. Studies on the 2009 swine influenza pandemic showed a significant increase in health-related fears [28-31]. Generally, health anxiety is characterized by excessive fears and worries about having, contracting, or developing a serious disease. Bleichhard and Hiller [32] found that a point prevalence of about 6% of the German population suffers from health anxiety disorders, which is in line with results from other population samples [33,34]. Parents may play a crucial role in transmitting health beliefs and related behavior to their children [35]. Contemporary research has provided some evidence of the interconnected anxiety of parents and their children during pandemics [17,36], which could prompt a parent-based intervention approach to simultaneously address fears and burden in families.

In this context, less is known about the relationship between health anxiety and family variables (eg, functioning or intimate partner relationship distress). Although research on general anxiety provides evidence of associations with relational variables [37-40], they are rarely assessed together with health anxiety inventories. Anxiety-related psychopathologies often but not necessarily include worries about health [41]. Although a few studies have examined the connection between health anxiety of children and family functioning [42,43], less research has focused on health anxiety in parents, particularly in the general population. Understanding the contribution of family and couple functioning in relation to health anxiety in the general population is particularly relevant in the context of the current pandemic.

The current body of literature for measuring parental distress, parental burden, or the quality of the caregiver-child relationship is extensive, with a long history of research [44,45]. However, there is a need to assess anxiety related to the COVID-19 pandemic among parents. To our knowledge, only one scale was developed to assess parenting during a pandemic [46]; however, it has not yet been validated in a parent sample. In terms of assessing COVID-19–related stress and anxiety, Ransing and colleagues [47] provided an overview of recently published scales. They identified five different scales in the literature up to May 15, 2020: the Fear of COVID-19 Scale (FCV-19S) [48], the Coronavirus Anxiety Scale (CAS) [49], the Obsession with COVID-19 Scale [50], the COVID Stress Scales (CSS) [51], and the Perception of Threat from COVID-19 [52]. Validation of these scales among parent samples has yet to be conducted as far as we are aware.

Stressors and needs within families with young children may differ from those of other household types, where childcare obligations or homeschooling can be serious challenges that are unique to parents [27,53]. Calls for initial measures for enhancing family-based interventions and sheltering vulnerable groups have been noted [21,54,55]. From a public health perspective, valid measurements for early detection of COVID-19–related anxiety among parents at risk may be useful for epidemiological studies as well as to identify parents in need of early intervention support through health care services or other social services [56,57].

### Objectives

The objectives of the current study are twofold: (1) to compare the distribution, validity, and reliability of four different COVID-19 anxiety and distress scales, namely the FCV-19S, CAS, PAS, and the *Danger* subscale of the CSS; (2) to perform an exploratory factor analysis (EFA) of all four scales to identify the most promising brief unidimensional scale that can be used efficiently for research and practice among parent samples. The included items should have sufficient variance in the sample, ability to detect symptoms in both mothers and fathers, moderate to high item discrimination, and associations with related constructs. We hypothesized that the COVID-19 anxiety measures would moderately correlate with trait health anxiety, show small to moderate correlations with other measures of family functioning (ie, parental stress and general family functioning), and show weak associations with intimate partner relationship satisfaction in a cross-sectional sample based on past literature on anxiety and families reviewed above.

## Methods

### Participants

A total of 1526 individuals started the web-based survey, resulting in a final sample of 515 parents after data cleaning (see below). Participants were predominantly mothers (465/515, 90.3%) with a university degree (307/515, 59.6%). Most of the 515 participants had German (312, 60.6%) or Austrian (177, 34.4%) citizenship. A share of 19.8% participants (102/515) came from Carinthia. The parents were aged 18-58 years (mean 34.95 years, SD 5.39). The majority were employed (285/515, 55.3%) or worked in the household (180/515, 34.9%). In terms of family status, 27.4% participants (141/515) were unmarried and 68.5% (353/515) were married. At the time of the survey, 94.4% participants (486/515) were in a partnership. Four participants (0.8%) stated that they had confirmed COVID-19 infection. More detailed demographics are described in Table 1.

**Table 1:** Sociodemographic characteristics of the study participants (N=515), n (%). The average age of the participants was 34.95 years (SD 5.39).

Characteristic		Value
**Gender**		
	Female	465 (90.3)
	Male	50 (9.7)
**Nationality**		
	Austria	177 (34.4)
	Germany	312 (60.6)
	Switzerland	6 (1.2)
	Other	20 (3.8)
**Marital status**		
	Unmarried	141 (27.4)
	Married	353 (68.5)
	Divorced	21 (4.1)
In a relationship^a^		486 (94.4)
**Number of children**		
	1	206 (40.0)
	2	239 (46.4)
	3	55 (10.7)
	4	10 (1.9)
	5 or more	5 (1.0)
Currently pregnant^a^		22 (4.3)
**Educational level**		
	No degree	1 (0.2)
	Lower secondary	30 (5.8)
	Higher secondary	68 (13.2)
	High school	109 (21.2)
	University	307 (59.6)
**Employment**		
	Employed	285 (55.3)
	Working in household	180 (34.9)
	Other	50 (9.8)

^a^Reflects the number and percentage of participants answering “yes” to this question.

### Survey Procedure

Participants were recruited on the web during a 6-week period (June 29 to August 9, 2020), mainly via social media in parenting or child-related Facebook groups and message boards. The evaluation of the HTTP referers showed that half of the final sample (50.48%) found the survey through Facebook. In addition, more than 4000 kindergartens and parent-child centers in Germany, Austria, and Switzerland were contacted electronically and asked to distribute the link of the web-based survey. Participants were required to be ≥18 years of age and the parent of at least one child aged between 0 and 6 years; participants were excluded if they or their children had any chronic or acute diseases. This study was part of a larger study focused on understanding parental search behaviors for health information, and the inclusionary criteria were required for the purposes of the overall study. The survey took an average of 22 minutes to complete. Participants were offered the chance to win ten vouchers in the amount of €10 (US $11.91) at the end of the survey. They were provided with a separate link to enter the raffle that could not be connected with the data from the study, which was anonymous. The study was approved by the Institutional Review Board of the University of Klagenfurt. Informed consent was obtained before data were collected.

### Data Cleaning

Prior to data analysis, the dataset of 1526 entries was cleaned in two waves. First, 978 participants (64.1%) failed to fill out the entire questionnaire. The majority of these participants ended the survey during or immediately after the demographics section (544/978). Only five participants dropped out during the COVID-19 items, which were presented on the last five pages. Second, 53 participants were excluded because they either stated that they had no children (n=5), or their youngest child was older than six years (n=48). This resulted in a final sample of 515 parents.

### Translation

All COVID-19 scales were only available in English and were thus translated into German by one author (CK) and an American Studies student using the translation-back-translation procedure. To further ensure quality, two psychology doctoral students subsequently checked the correctness of the translations independently. Based on this check, some minor changes were made to individual items to improve readability and precision. Some of the questionnaires used different spellings for COVID-19 (eg, coronavirus-19, COVID-19, virus). We decided to use the term “Covid-19” consistently, as this spelling is common in German-speaking countries. All translated questionnaires can be found in Multimedia Appendix 1.

### Data Analysis Strategy

All descriptive and correlational analyses were performed using SPSS version 25 (IBM Corporation). A two-tailed *P* value <.05 was considered statistically significant. We followed the Cohen [58] interpretation guidelines for Pearson correlations, with *r*=0.10 considered to be a small correlation, *r*=0.30 a medium correlation, and *r*=0.50 a large correlation. For the EFA model fit, the root mean square error of approximation (RMSEA) was calculated with JASP version 0.11.1 [59]. The RMSEA fit index was interpreted according to Browne and Cudeck [60].

In addition, we applied an item response theory (IRT) approach to provide measures for item discriminability and difficulty. The graded response model by Samejima [61] was used. This model is an extension of the two-parameter logistic model that is applicable for ordered polytomous variable data (eg, Likert scales). A sample size of N=500 is recommended for accurate parameter estimation [62]. Marginal maximum likelihood estimation [63] was used for estimation of the parameters. We calculated item discrimination (alpha) and item difficulty (beta) for each scale separately based on the initial proposed unidimensional factor structures of COVID-19–related anxiety scales. As a result, the PAS was only considered with the subscale “disease anxiety” for the IRT analysis. All other scales were included in the analysis in their entirety, as they were proposed to measure one factor. According to the guidelines of Baker and Kim [64], we interpreted alpha values ≤0.64 as low item discrimination, values between 0.65 and 1.34 as moderate, and values ≥1.35 as high. IRTPRO software was used to estimate the parameters of the IRT models [65].

### Measures

#### The One-Item Covid-Fear Scale

The One-Item Covid-Fear scale (Covid-F) was developed for this study. The item assessed fear of COVID-19 (“How do you rate your fear of the coronavirus (Covid-19)?”) based on a 10-point Likert-scale (1-10), with a higher score indicating greater fear.

#### The FCV-19S

The FCV-19S, developed by Ahorsu et al [48], is a 7-item inventory using a 5-point Likert scale with scores between 7 and 35. The higher the score, the higher the fear of COVID-19. The scale showed good internal consistency (α=.82). Moderate correlations with depression (*r*=0.42) and anxiety (*r*=0.51) were reported. Validation studies were performed with samples from Russia and Belarus [66], Italy [67], Bangladesh [68], Turkey [69], Saudi Arabia [70], Israel [71], India [72], Greece [73], the United States [74], Spain [75], Japan [76], Cuba [77], and Mexico [78]. Overall, the FCV-19S has shown robust psychometric properties across validation studies; the findings predominantly support a unidimensional factor structure.

#### The CAS

The CAS, developed by Lee [49], is a short 5-item screening instrument that assesses common physiological anxiety symptoms related to COVID-19 over the previous two weeks: dizziness, sleep disturbance, tonic immobility, appetite loss, and abdominal distress. Confirmatory factor analysis (CFA) indicated a single factor structure of the coronavirus anxiety construct. The scale showed excellent internal consistency (α=.93) in the initial validation study. Scores can range between 0 and 20. Associations were found with COVID-19 diagnosis, functional impairment, and maladaptive coping strategies, but not with history of anxiety. The suggested cutoff score (≥9) identified burdened adults with 90% sensitivity and 85% specificity for dysfunctional levels of COVID-19–related anxiety. Validation studies were performed in Turkey [79] and Bangladesh [80].

#### The PAS

The PAS, developed by McElroy et al [81], is a 7-item scale for assessing anxiety experienced during a pandemic. In a validation study, 4793 parents with children aged between 4 and 16 years were included. Total scores can range between 0 and 28. EFA revealed a two-factor solution with four items regarding contracting and transmitting the virus (disease anxiety) and three items concerning worries about consequences of the pandemic (consequence anxiety). This factor structure was verified with CFA in the other half of the sample. Internal consistency across all items was acceptable (α=.70). Moderate correlation was found with a subset of items of the Depression, Anxiety, and Stress Scale [82]. The PAS was also tested in a sample of medical students and residents in the United Kingdom [83].

#### The CSS

The CSS, developed by Taylor et al [51], is a 36-item inventory that consists of five subscales: danger and contamination fears, socioeconomic consequences, xenophobia, traumatic stress symptoms, and compulsive checking related to COVID-19. Initially, the scale was validated in a Canadian and US sample. The internal consistencies varied from α=.83 to α=.95 for the different subscales, and the subscales were moderately to highly correlated.

In the initial 6-factor solution, the scales of danger and contamination were divided into 2 subscales; however, due to high cross-loadings, they were combined a posteriori. For our study, we only used the 6 items of the danger subscale. This subscale includes relational items that seem especially relevant for parents (eg, “I am worried that I can’t keep my family safe from the virus”). Further studies have been conducted in an additional US and Canadian sample [84] and in the Philippines [85].

### Validity Measures

#### The Modified Short Health Anxiety Inventory

The Modified Short Health Anxiety Inventory (mSHAI), developed by Bailer et al [86], is a 14-item test instrument for the measurement of trait health anxiety as a single construct. A meta-analysis has shown that the original Short Health Anxiety Inventory by Salkovskis et al [87] is a valid, reliable, and useful instrument for assessing health anxiety in clinical and non-clinical samples [88]. In contrast to the original inventory by Salkovskis et al [87], the mSHAI has a simpler response format on a 5-point Likert scale. Total scores range between 0 and 56. The mSHAI showed excellent internal consistency in our sample (α=.94). We expected COVID-19 anxiety scales to only weakly or moderately correlate with this measure of trait health anxiety because the COVID-19 pandemic is uniquely impacting parents, who otherwise would have low levels of health anxiety.

#### The Couple Satisfaction Index

The Couple Satisfaction Index (CSI), developed by Funk and Rogge [89], is a widely used measurement in research and practice for relationship satisfaction. The basic version contains 32 items (CSI-32); however, the short version with 16 items (CSI-16) demonstrates strong psychometric properties and precision in detecting couple satisfaction compared to other measures. Total scores can range between 0 and 81. Scores below the recommended cutoff score of 51.5 indicate substantial relationship distress. In our sample, the Cronbach coefficient of the CSI-16 was excellent, with α=.97.

#### Parental Stress Scale

The Parental Stress Scale (PSS), developed by Berry and Jones [90], is an 18-item scale for assessing child-related burden in mothers and fathers. Scores range from 18 to 90. A higher score indicates a higher level of parental stress. Factor analysis identified four dimensions: parental rewards, parental stressors, loss of control, and parental satisfaction. Despite some discord in the literature about the initial factor structure [91], the PSS is a psychometrically robust and widely used measurement in both clinical and nonclinical samples. The internal consistency in the present sample was good, with Cronbach α=.86.

#### The General Functioning Scale of the Family Assessment Device

The General Functioning Scale (GFS) [92] is a 12-item subscale of the McMaster Family Assessment Device [93] to assess family functioning. Parents evaluate statements about family life on a 4-point Likert scale. The total score is then divided by 12 to give the overall functional level. A score of 1.0 indicates healthy family functioning, while a score of 4.0 represents extremely poor family functioning. Byles et al [92] recommended 2.17 as a cutoff score to detect dysfunctional families. The measure is correlated with a variety of other measures of problems, including alcohol abuse, marital distress, partner violence, and parental separation. In our sample, the GFS showed good internal consistency (α=.87).

## Results

### Sample Descriptive Statistics and Gender Differences

Overall, 27.4% respondents (133/486) scored below the distress cutoff of the CSI-16, indicating couple dissatisfaction. In the measure of family functioning, 17.5% respondents (90/515) were identified as reporting problematic family functioning. There was a significant difference in trait health anxiety (mSHAI) scores for mothers and fathers (t_513_=2.30, *P*=.02); mothers had higher scores. However, there were no differences between mothers and fathers regarding COVID-19–related fear (Covid-F) (t_513_=0.49, *P*=.62). No significant gender differences were found for couple satisfaction, parenting stress, or family functioning (all *P*<.05).

Table 2 shows the range, mean, SD, score range, skewness, and kurtosis for all scales. With the exception of the PAS, all COVID-19–related scales were right skewed. None of the scales were normally distributed as assessed by Shapiro-Wilk test, *P*≤.001. In particular, the CAS showed the least variance. More than three quarters of the participants had zero variance (no endorsed symptoms) on this scale (402/515, 78.1%).

Next, independent sample *t* tests were conducted to compare the total scores of the COVID-19 scales between mothers and fathers. There were no significant differences in scores on the CAS (t_513_=1.03, *P*=.30), PAS (t_513_=−0.28, *P*=.77), or CSS-D (t_513_=−0.08, *P*=.93). There was a significant difference in scoring for the FCV-19S, with higher scores among mothers than fathers (t_513_= 2.98, *P*=.003).

**Table 2:** Descriptive statistics of the scales (N=515 for all scales, except CSI-16, for which n=486).

Scale	Range of the scale	Mean (SD)	Score range	Skewness	Kurtosis	Shapiro-Wilk test	*P* value of the Shapiro-Wilk test
mSHAI^a^	0-56	13.99 (10.66)	0-56	1.12	1.47	0.91	<.001
Covid-F^b^	1-10	4.10 (2.25)	1-10	0.50	−0.67	0.93	<.001
FCV-19S^c^	7-35	13.39 (4.96)	7-35	0.91	0.80	0.93	<.001
CAS^d^	0-20	0.67 (1.80)	0-15	3.96	18.89	0.43	<.001
PAS^e^	0-28	10.63 (5.29)	0-25	0.13	−0.48	0.98	<.001
CSS-D^f^	0-24	6.07 (5.47)	0-24	0.86	0.01	0.90	<.001
CSI-16^g^	0-81	58.81 (17.12)	3-81	−1.03	0.50	0.91	<.001
PSS^h^	18-90	39.21 (8.99)	18-73	0.33	0.06	0.99	.003
GFS^i^	1-4	1.71 (0.52)	1-3.75	0.10	1.02	0.92	<.001

^a^mSHAI: modified Short Health Anxiety Inventory.

^b^Covid-F: One-Item Covid-Fear scale.

^c^FCV-19S: Fear of COVID-19 Scale.

^d^CAS: Coronavirus Anxiety Scale.

^e^PAS: Pandemic Anxiety Scale.

^f^CSS-D: COVID Stress Scales–Danger subscale.

^g^CSI-16: 16-item Couple Satisfaction Index.

^h^PSS: Parenting Stress Scale.

^i^GFS: General Functioning Scale.

### Reliability

Internal consistencies for each of the four scales are presented in Table 3. All four scales showed at least acceptable consistency (unstandardized Cronbach α>.70) [94]. Inter-item average correlations were between 0.30 and 0.63.

**Table 3:** Reliability of the COVID-19–related anxiety and distress scales (N=515).

Scale	Cronbach α	McDonald ω	Gutmann λ6	Inter-item correlation
FCV-19S^a^	.87	0.88	0.89	0.52
CAS^b^	.83	0.84	0.82	0.51
PAS^c^	.73	0.75	0.78	0.30
CSS-D^d^	.91	0.91	0.91	0.63

^a^FCV-19S: Fear of COVID-19 Scale.

^b^CAS: Coronavirus Anxiety Scale.

^c^PAS: Pandemic Anxiety Scale.

^d^CSS-D: COVID Stress Scales–Danger subscale.

### Correlations With COVID-19 Anxiety Scales

Prior to analyzing their validity, the correlations of the scales with the demographic characteristics of the participants and the COVID-19 scales were examined. The parents’ age, years in a relationship, age of the youngest child, and number of children were not significantly correlated with FCV-19S, CAS, PAS, or CSS-D (all *P*>.05).

To investigate the convergent validity, we examined bivariate correlations between the four COVID-19 anxiety scales (Table 4). Moderate to high correlations of the four COVID-19 anxiety scales were found, ranging between *r*=0.36 and *r*=0.65. Except for the CAS, all scales had moderate correlations with the One-Item Covid-Fear scale, indicating convergent validity. Small to medium positive correlations were found between health anxiety as a trait (mSHAI) and the different COVID-19 scales, ranging from *r*=0.21 to *r*=0.38.

**Table 4:** Pearson correlations for COVID-19 anxiety scales and other measures of anxiety and family variables. N=515 for all scales, except CSI-16, for which n=486.

Variable		mSHAI^a^	Covid-F^b^	FCV-19S^c^	CAS^d^	PAS^e^	CSS-D^f^	CSI-16^g^	PSS^h^	GFS^i^
**mSHAI**										
	*r*	1	0.19	0.38	0.28	0.21	0.26	−0.13	0.19	0.15
	*P* value	—^j^	<.001	<.001	<.001	<.001	<.001	.002	<.001	.001
**Covid-F**										
	*r*	0.19	1	0.71	0.32	0.56	0.69	−0.03	0.09	0.05
	*P* value	<.001	—	<.001	<.001	<.001	<.001	.50	.03	.23
**FCV-19S**										
	*r*	0.38	0.71	1	0.51	0.61	0.65	−0.07	0.17	0.13
	*P* value	<.001	<.001	—	<.001	<.001	<.001	.08	<.001	.002
**CAS**										
	*r*	0.28	0.32	0.51	1	0.36	0.40	−0.12	0.15	0.16
	*P* value	<.001	<.001	<.001	—	<.001	<.001	.007	<.001	<.001
**PAS**										
	*r*	0.21	0.56	0.61	0.36	1	0.61	−0.07	0.25	0.11
	*P* value	<.001	<.001	<.001	<.001	—	<.001	.10	<.001	.007
**CSS-D**										
	*r*	0.26	0.69	0.65	0.40	0.61	1	−0.05	0.19	0.14
	*P* value	<.001	<.001	<.001	<.001	<.001	—	.27	<.001	.001
**CSI-16**										
	*r*	−0.13	−0.03	−0.07	−0.12	−0.07	−0.05	1	−0.31	−0.80
	*P* value	.002	.50	.08	.007	.10	.27	—	<.001	<.001
**PSS**										
	*r*	0.19	0.09	0.17	0.15	0.25	0.19	−0.31	1	0.35
	*P* value	<.001	.03	<.001	<.001	<.001	<.001	<.001	—	<.001
**GFS**										
	*r*	0.15	0.05	0.13	0.16	0.11	0.14	−0.80	0.35	1
	*P* value	.001	.23	.002	<.001	.007	.001	<.001	<.001	—

^a^mSHAI: modified Short Health Anxiety Inventory.

^b^Covid-F: One-Item Covid-Fear scale.

^c^FCV-19S: Fear of COVID-19 Scale.

^d^CAS: Coronavirus Anxiety Scale.

^e^PAS: Pandemic Anxiety Scale.

^f^CSS-D: COVID Stress Scales–Danger subscale.

^g^CSI-16: 16-item Couple Satisfaction Index.

^h^PSS: Parenting Stress Scale.

^i^GFS: General Functioning Scale.

^j^—: not applicable.

### Validity Analyses With COVID-19 Anxiety Scales and Family Measures

As hypothesized, small positive correlations between the four COVID-19 scales were found with parenting stress (*r*=0.15-0.25) and general family functioning (*r*=0.11-0.16). No significant associations were found between the COVID-19 scales and couple satisfaction, except in the case of the CAS, which showed a small negative correlation with couple satisfaction. Among family measures, all scales correlated at least moderately.

### EFA of All COVID-19 Anxiety Scales

We performed an additional EFA on all 25 items of the COVID-19 scales to examine the overall similarity of the constructs (Table 5). The Kaiser-Meyer-Olkin measure of sampling adequacy was 0.93. The Bartlett test of sphericity was significant (*P*<.001). Varimax (orthogonal) rotation was applied. The Kaiser criteria [95] and scree plot retained a 5-component solution with initial eigenvalues of 8.49, 2.62, 1.83, 1.48, and 1.01, which accounted for 67.10% of the total variance. The model showed acceptable fit (RMSEA=0.068, 90% CI 0.061-0.073, Tucker-Lewis index=0.909).

The first factor included eight total items from the CSS (CSS-1 and CSS-4), FCV-19S (FCV-1, FCV-2, and FCV-5), and PAS (PAS-1, PAS-2, and PAS-4) with loadings higher than 0.40, and it accounted for 39.24% of the total variance. This factor represented COVID-19–related fear of infection. The second factor accounted for 10.5% of the total variance and was formed by the six items of the CSS-D. However, two items had cross-loadings on the first factor. One item was nearly identical to another (CSS-1 and FCV1), and the other item relates to protecting one’s family from the virus. The third factor explained 7.32% of the variance and contained six items without clear content focus, including fear of dying, nervousness about news on social media, physical symptoms of anxiety, and insomnia. One item of the PAS, on fear of leaving the house, was also loaded on this factor. Furthermore, the five items of the CAS were all loaded uniquely on the fourth factor, representing physical symptoms of anxiety and explaining an additional 5.94% of the variance. Surprisingly, these items did not load sufficiently with those of the FCV-19S on physical anxiety symptoms. Finally, three items of the PAS (PAS5, PAS5, and PAS7) formed the fifth factor regarding the socioeconomic consequences of COVID-19, explaining 4.07% of the total variance.

**Table 5:** Exploratory factor analysis of the CAS, CSS, FCV-19S, and PAS scales. The varimax rotation method was applied.

Scale item	Factor 1	Factor 2	Factor 3	Factor 4	Factor 5	Uniqueness
CAS^a^_CAS1	—^b^	—	—	.666	—	.414
CAS_CAS2	—	—	—	.665	—	.449
CAS_CAS3	—	—	—	.787	—	.312
CAS_CAS4	—	—	—	.798	—	.324
CAS_CAS5	—	—	—	.766	—	.385
CSS^c^_CSS1	.610	.436	—	—	—	.360
CSS_CSS2	—	.692	—	—	—	.312
CSS_CSS3	—	.843	—	—	—	.189
CSS_CSS4	.468	.694	—	—	—	.244
CSS_CSS5	—	.826	—	—	—	.206
CSS_CSS6	—	.711	—	—	—	.339
FCV-19S^d^_FCV1	.687	—	—	—	—	.262
FCV-19S_FCV2	.727	—	—	—	—	.334
FCV-19S_FCV3	—	—	.765	—	—	.347
FCV-19S_FCV4	—	—	.591	—	—	.392
FCV-19S_FCV5	.431	—	—	—	—	.425
FCV-19S_FCV6	—	—	.813	—	—	.220
FCV-19S_FCV7	—	—	.818	—	—	.212
PAS^e^_PAS1	.754	—	—	—	—	.225
PAS_PAS2	.715	—	—	—	—	.301
PAS_PAS3	—	—	.479	—	—	.525
PAS_PAS4	.648	—	—	—	—	.522
PAS_PAS5	—	—	—	—	.746	.354
PAS_PAS6	—	—	—	—	.864	.222
PAS_PAS7	—	—	—	—	.796	.350

^a^CAS: Coronavirus Anxiety Scale.

^b^Factor loadings below .40 are omitted from the table to improve readability.

^c^CSS: COVID Stress Scales.

^d^FCV-19S: Fear of COVID-19 Scale.

^e^PAS: Pandemic Anxiety Scale.

### IRT Analysis

Based on the results of the EFA, the overall set of items did not appear to have a common unidimensional latent structure. In addition, the response format options were not identical for all questionnaires. Therefore, we conducted an analysis for each scale separately. Parameter estimation for item discrimination (ie, slopes) and item difficulty (ie, thresholds) can be found in Table 6. Characteristic curves for the individual items are available in Multimedia Appendix 2.

**Table 6:** Graded response model parameter estimates for the CAS, CSS-D, FCV-19S, and PAS (N=515).

Item		Discrimination	Difficulty			
		α (SE)	*β_1_*	*β_2_*	*β_3_*	*β_4_*
**CAS^a^**						
	CAS-1	3.01 (0.39)	1.49	2.18	2.86	N/A^b^
	CAS-2	2.66 (0.41)	1.35	2.00	2.98	3.52
	CAS-3	3.98 (1.97)	1.26	1.91	2.54	3.09
	CAS-4	4.03 (1.05)	1.74	2.29	2.92	N/A
	CAS-5	3.35 (0.56)	1.82	2.30	2.72	3.02
**CSS-D^c^**						
	CSS-D1	1.97 (0.16)	−0.34	0.72	1.75	3.04
	CSS-D2	2.79 (0.24)	0.01	0.90	1.60	2.57
	CSS-D3	3.86 (0.35)	0.08	0.75	1.32	2.36
	CSS-D4	3.66 (0.31)	−0.62	0.26	0.88	1.88
	CSS-D5	3.90 (0.34)	−0.34	0.42	0.96	1.77
	CSS-D6	2.57 (0.22)	0.06	0.90	1.58	2.58
**FCV-19S^d^**						
	FCV-19S-1	2.24 (0.19)	−0.83	0.12	1.21	2.52
	FCV-19S-2	1.91 (0.16)	−1.42	−0.60	0.22	1.83
	FCV-19S-3	2.68 (0.27)	0.65	1.75	2.86	3.60
	FCV-19S-4	2.48 (0.23)	0.29	1.05	1.83	2.70
	FCV-19S-5	2.26 (0.19)	−0.31	0.58	1.34	2.56
	FCV-19S-6	4.40 (0.57)	0.66	1.55	2.26	2.98
	FCV-19S-7	4.47 (0.59)	0.70	1.42	1.92	2.97
**PAS^e^**						
	PAS-1	4.19 (0.76)	−0.72	−0.00	0.65	1.95
	PAS-2	4.10 (0.77)	−1.20	−0.38	0.17	1.54
	PAS-3	1.49 (0.16)	0.69	2.17	3.51	N/A
	PAS-4	1.30 (0.12)	−1.06	−0.11	0.74	2.67

^a^CAS: Coronavirus Anxiety Scale. Difficulty parameters for responses on a 5-point Likert scale: β_1_ (from “not at all” to “rare, less than a day or two”), β_2_ (from “rare, less than a day or two” to “several days”), β_3_ (from “several days” to “more than 7 days”), and β_4_ (from “more than 7 days” to “nearly every day over the last 2 weeks”).

^b^N/A: not applicable (*β*4 could not be calculated for CAS-1, CAS-4, or PAS-3 due to the unused item response range).

^c^CSS-D: COVID Stress Scales–Danger subscale. Difficulty parameters for responses on a 5-point Likert scale: β_1_ (from “Not at all” to “slightly”), β_2_ (from “slightly” to “moderately”), β_3_ (from “moderately” to “very”), and β_4_ (from “very” to “extremely”).

^d^FCV-19S: Fear of COVID-19 Scale. Difficulty parameters for responses on a 5-point Likert scale: β_1_ (from “strongly disagree” to “disagree”), β_2_ (from “disagree” to “neither agree nor disagree”), β_3_ (from “neither agree nor disagree” to “agree”), and β_4_ (from “agree” to “strongly agree”).

^e^PAS: Pandemic Anxiety Scale. Difficulty parameters for responses on a 5-point Likert scale: β_1_ (from “strongly disagree” to “disagree”), β_2_ (from “disagree” to “neither agree nor disagree”), β_3_ (from “neither agree nor disagree” to “agree”), and β_4_ (from “agree” to “strongly agree”).

Item discrimination was high for all items except for the PAS-4 item on worry about transferring the infection to someone else, which had a moderate level of discrimination. High alpha values in all scales indicate that the items were able to discriminate parents with a high latent trait from those with a low latent trait. With respect to item difficulty, only the CAS provided exclusively positive threshold parameters, suggesting that these items perform best when measuring people with higher levels of the latent trait.

**Figure 1:**
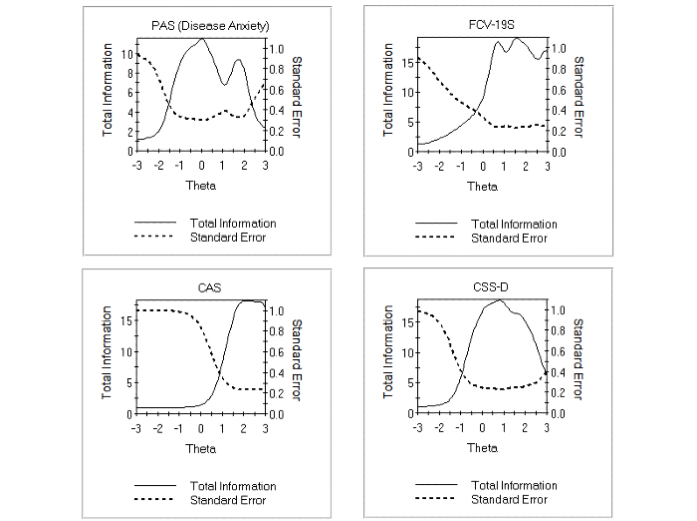
Total information functions for the PAS, FCV-19S, CAS and CSS-D scales. CAS: Coronavirus Anxiety Scale; CSS-D: COVID Stress Scales–Danger subscale; FCV-19S: Fear of COVID-19 Scale; PAS: Pandemic Anxiety Scale.

The test information function of each scale is presented in Figure 1. All scales have the tendency to provide more information between 0 and +2 SDs than between 0 and −2 SDs. The CAS provides insufficient information for parents with scores lower than the mean. The PAS and CSS-D achieved good accuracy between the mean and ±1 SD. High values of the latent trait with +3 SDs were measured accurately with CAS and FCV-19S, but less precise with PAS or CSS-D. Detailed item information function values at different theta levels can be found in Table 7.

**Table 7:** Item information function values for each scale at θ values between –2.4 and 2.4.

Scale	θ value						
	−2.4	−1.6	−0.8	0.0	0.8	1.6	2.4
PAS^a^	1.37	4.16	9.85	11.48	7.94	9.14	4.59
FCV-19S^b^	1.62	2.91	5.02	9.07	18.34	19.13	15.85
CAS^c^	1.00	1.00	1.04	1.43	5.54	16.21	17.99
CSS-D^d^	1.11	1.94	8.46	16.73	18.64	16.42	12.28

^a^PAS: Pandemic Anxiety Scale.

^b^FCV-19S: Fear of COVID-19 Scale.

^c^CAS: Coronavirus Anxiety Scale.

^d^CSS-D: COVID Stress Scales–Danger subscale.

### Additional Analysis of Selected Items of the PAS Subscale

We selected items for further investigation based on the analysis of distributions, variance in the sample, exploratory factor analysis, and IRT analysis. This resulted in three items on infection worries regarding oneself (PAS-1) as well as family and friends (PAS-2), and the possibility of spreading the virus to someone else (PAS-4). These were all obtained from the PAS subscale on disease anxiety. We did not consider the other items in the first factor (Table 5) due to substantial cross-loadings above .4 on other factors or gender differences in scoring (*P*<.05).

The items of the other factors were not considered for the following reasons. The second factor (see Table 5) consists mainly of items regarding the role of the health care system and information provided on containment. Further, the third factor lacked coherence due to high variability of content with different cognitive and behavioral dimensions. Another issue was the different mean scores for mothers and fathers (*P*<.01) on some of these items (FCV-5, FCV-6, and FCV-7), which indicate gender-specific differences. Moreover, although all items of the CAS loaded on an anxiety-related fourth factor, they showed insufficient variance in the sample, with 78.1% of participants (402/515) not endorsing a single item. An additional IRT analysis revealed a lack of sufficient test information for parents with scores equal to or lower than the mean. Finally, the items of the fifth factor (PAS5, PAS6, and PAS7) referred only to socioeconomic consequences of the COVID-19 pandemic without being related to anxiety.

Accordingly, we examined the psychometric properties of the 3-item PAS scale assessing disease anxiety. Scores can range between 0 and 12 (mean 5.25, SD 3.06). The scale showed acceptable internal consistency (α=.79). There was no significant scoring difference for mothers and fathers (t_513_=−0.79, *P*=.42). Parents with elevated health anxiety had higher scores (t_513_= −2.70, *P*=.007). High correlations were found with the One-Item Covid-Fear scale (*r*=0.69), the FCV-19S (*r*=0.79), the PAS (*r*=0.66), and the CSS-D (*r*=0.70). Small to moderate correlation was found with the CAS (*r*=0.28), trait health anxiety (*r*=0.18) and parenting stress (*r*=0.15; all *P*<.001). Nonsignificant correlations were found with age (*r*=0.04, *P*=.27), length of partnership (*r*=0.00, *P*=.88), age of the youngest child (*rho*=0.01, *P*=.79), couple satisfaction (*r*=0.00, *P*=.98) and family functioning (*r*=0.05, *P*=.20).

## Discussion

### Principal Findings

Our aim was to evaluate various existing scales for COVID-19–related anxiety and fear (ie, basal anxiety regarding the COVID-19 pandemic and the infection itself, out of pre-existing scales). In our sample, all four scales (the FCV-19S, PAS, CAS, and CSS-D) had adequate psychometric properties. However, exploratory factor analysis revealed that different facets of anxiety and worries were measured across the scales. Based on our classical test theory and IRT analysis, the PAS subscale on disease anxiety for assessing COVID-19–related anxiety seems to be appropriate as a brief scale. However, factor analysis suggests using only the items PAS-1 (i.e. self-infection), PAS-2 (ie, infection of family and friends), and PAS-4 (ie, spreading of infection) for unidimensional assessment. We were able to show that these three items are psychometrically sound for covering general infection anxiety related to COVID-19 in parents. Nonetheless, all the investigated inventories had strengths, and the selection of which scale to use may be dependent on the sample in which it will be used (eg, clinical vs nonclinical, parent vs nonparent, or families with toddlers vs families with older children).

Although the CAS has a one-dimensional structure without cross-loadings on other factors, floor effects were found for three-quarters of the participants (ie, zero variance). This inventory assesses distressing bodily symptoms and may not capture general COVID-19–related stress among community samples; however, it may be suitable for clinical samples. In the fifth edition of the *Diagnostic and Statistical Manual of Mental Disorders*, the constructs of somatic symptom disorder (F45.1) and illness anxiety disorder (F45.21) replaced hypochondria [96,97]. We suspect that the CAS best detects whether parents report a somatic expression of COVID-19–related anxiety, but not necessarily whether their fear is predominantly cognitive. Interestingly, the FCV-19S contains items on somatic symptoms of anxiety, such as clammy hands and tachycardia, which did not load on the same factor as the CAS items.

The initially proposed two-factor structure of the PAS was partially replicated with disease anxiety and consequence anxiety as two latent factors. The item assessing “worries about leaving the house” no longer loaded on either factor and can be explained by timing of the data collection in the original study. McElroy et al [81] collected data early during the pandemic outbreak in April 2020, when lockdowns were in effect. This suggests that the influence of COVID-19–related anxiety items may change when perception of risk situations changes over time in society. It may be important for longitudinal studies on understanding COVID-19–related anxiety to include and test items that are relevant regardless of changes in lockdowns and public health measures.

Further, we observed lower means for all items and scales than in other studies on these measures [48,49,81]. It should be noted that the overall level of fear is probably strongly dependent on the time of the survey, the country of assessment, local closeness to infection clusters, and media reporting. At the time of our survey period, in July 2020, the number of infections in German-speaking countries was relatively stable, with greater infection clusters in a subset of settings [98]. In contrast, the validation studies [48,49,51,81] all took place between March and April 2020, at the onset of the pandemic outbreak, when there was a high level of uncertainty regarding the course of the pandemic.

In addition, small to moderate bivariate correlations between health anxiety as a trait (measured with the mSHAI) and the instruments raised questions about COVID-19–related fear and its association with health anxiety. The One-Item Covid-Fear scale had a Pearson *r* value of 0.19 with mSHAI. The correlations of mSHAI with the CAS, PAS, and CSS-D scales were in the range of *r*=0.20-0.28. This suggests that pandemic-related health anxiety is distinct from trait health anxiety and should be assessed separately.

There may be several explanations for the small associations between COVID-19–related anxiety and health anxiety. Previous studies found different antecedents for COVID-19–related fears: fear about economic consequences, fear of new measures, fear of health care collapse, fear of illness, fear of death, or fear of spreading the virus to risk groups [99-102]. We assume that these fears can appear independently from each other. Not all of these fears are health-related; therefore, they are not necessarily linked to an individual’s own health anxiety [103].

In addition, the construct of trait health anxiety is based on relatively stable negative health-related cognitions and preoccupation with one's own health [96,104]. COVID-19–related anxiety may affect cognitions differently due to the public attention paid to the virus in the media, which places the focus on a public health level rather than on an individual health level. Similarly, COVID-19–related anxiety may be perceived more as a threat to an individual’s family than to their own health.

In our factor analysis, all items of the CSS-D loaded on a common unique factor that had cross loadings with the general COVID-19–related anxiety factor (see Table 5). We suspect that this perceived fundamental threat occurs regardless of health anxieties and is represented in this factor. For example, an early study on the H1N1 influenza pandemic from Jones and Salathé [29] found strong clustering of anxiety related to H1N1 influenza with anxiety over trauma. The operationalization of COVID-19–related anxiety as related to threat and traumatic event perception rather than health anxiety has implications for prevention and treatment (see [12,105]). The use of a traumatic stress framework was already noted during the previous H1N1 pandemic for families [31]; however, COVID-19–specific trauma research is needed [12].

A secondary goal of the study was to investigate the association between COVID-19–related anxiety and family variables (ie, couple satisfaction, family functioning, and parenting stress). We did not find significant associations between couple satisfaction and COVID-19 measures. Although there is some evidence of a link between couple distress and anxiety [37], findings related to general anxiety symptoms or disorders may not generalize to COVID-19–related anxiety, which is related to a population-level public health crisis. In addition, we suspect that the relationship between COVID-19–related anxiety and couple satisfaction may be moderated by other variables that were not assessed, such as social support or work stress [106].

As hypothesized, parenting stress and family functioning showed small correlations with COVID-19–related anxiety among parents. Intriguingly, neither the One-Item Covid-Fear scale (Covid-F) nor the PAS subscale on disease anxiety correlated with family functioning, although all other tested COVID-19 scales did. Certain families experienced chronic stress and anxiety from the pandemic, which one would expect to impact family well-being and functioning over time and should therefore be associated [107,108]. It is possible that the high education levels in our sample may have weakened the relationship between family functioning and COVID-19–related anxiety that might be seen among samples with a wider spectrum of socioeconomic status [109-111]. In contrast, the PSS correlated consistently with all COVID-19 scales. Parenting stress may be a better indicator of COVID-19–related impacts than a general family functioning measure, which may have a more distal relationship [107]. Especially, some items of the parental stress scale were highly relevant during the time of recruitment, with limited possibility of childcare offers (eg, “Having child(ren) leaves little time and flexibility in my life”), and may have captured the COVID-19–related burden. It is possible that prolonged exposure to increased parental stress would have an effect on worsening family functioning over time. Prospective designs are needed to best understand the impacts of COVID-19–related anxiety on the parental relationship, parenting stress, and family functioning.

Finally, more than one in four parents showed significant distress in their partner relationship, and almost every fifth family had poor family functioning. Parental stress was equally substantial across mothers and fathers. High numbers of burdened parents during the COVID-19 pandemic have been reported in other studies, along with serious warnings regarding increased violence potential in families [14,22,112]. We encourage policy makers to focus on families as an important societal functional unit. Initial support for burdened parents is urgently needed at all levels to mitigate the negative impact of COVID-19 on mental health in parents and children by providing public health education [113], offering positive parenting training and psychological support via telehealth [114,115], providing funding to mitigate economic hardship [116], strengthening couple relationships, and promoting general family functioning for building resilience [117].

### Limitations

The study is cross-sectional; thus, we cannot make any statements about causalities. All measures were translated into German and tested in a German-speaking sample. It is conceivable that there are language or country-specific differences. We excluded people with self-reported acute medical conditions, which means that the results are only generalizable to a sample of parents without medical conditions. It is possible that the relationship between health anxiety and COVID-19–related anxiety would be different in a sample of parents with acute or chronic medical conditions. Another possible limitation is that all parents were recruited on the web. Therefore, our results could be biased through self-selection [118] and overrepresentation of parents using social media. Further, more mothers participated than fathers; therefore, further validation work with fathers is needed.

### Conclusion

This study highlights how some of the existing scales on COVID-19–related anxiety measure different facets of pandemic-related anxiety among parents of young children. The differences across highlighted measures can serve as a guide for future selection of brief measures that assesses COVID-19–related anxiety among parents, which may be useful for future research. This study also highlights the associations between family variables and COVID-19–related anxiety, particularly in the case of parental stress. Future research should examine how anxiety can impact family relationships over time to better understand the potential impact of the pandemic on both mental health and family health. The results should also be replicated in other countries and cultures to best understand additional contextual factors.

## Appendix

Multimedia Appendix 1 Translations of the questionnaires.

Multimedia Appendix 2 Item response theory analysis (item characteristic curve, total information curve, and test characteristic curve).

## Abbreviations

CAS: Coronavirus Anxiety Scale
CFA: confirmatory factor analysis
Covid-F: One-Item Covid-Fear scale
CSI: Couple Satisfaction Index
CSS: COVID Stress Scales
CSS-D: COVID Stress Scales—Danger subscale
EFA: exploratory factor analysis
FCV-19S: Fear of COVID-19 Scale
GFS: General Functioning Scale
IRT: item response theory
mSHAI: Modified Short Health Anxiety Inventory
PAS: Pandemic Anxiety Scale
PCA: Principal Component Analysis
PSS: Parenting Stress Scale
RMSEA: root mean square error of approximation
