# Supplementary material for: Measuring COVID-19 Related Anxiety in Parents: Psychometric Comparison of Four Different Inventories
Source: JMIR Ment Health. 2020 Dec 3;7(12):e24507. doi: 10.2196/24507 (PMC7717922; doi:10.2196/24507)
Supplement: Multimedia Appendix 1 [file mental_v7i12e24507_app1.pdf]

Appendix

Translations:

Covid-F

1. Wie schätzen Sie ihre Angst vor dem Corona-Virus (Covid-19) ein?

FCV-19S

Antwortformat: Starke Ablehnung, Ablehnung, Weder Ablehnung noch Zustimmung, Zustimmung, Starke Zustimmung

1. Ich habe große Angst vor Covid-19.
2. Ich fühle mich unwohl bei dem Gedanken an Covid-19.
3. Ich bekomme schwitzige Hände, wenn ich an Covid-19 denke.
4. Ich habe Angst an Covid-19 zu sterben.
5. Wenn ich Nachrichten und Berichte über Covid-19 in sozialen Medien sehe, werde ich nervös oder ängstlich.
6. Ich kann nicht schlafen, da ich mir Sorgen mache, mich mit Covid-19 zu infizieren.
7. Mein Herz rast oder klopft, wenn ich daran denke, mich mit Covid-19 zu infizieren.

CAS

Instruktion: Wie oft haben Sie in den letzten zwei Wochen die folgenden Symptome erlebt?
Antwortformat: Überhaupt nicht, Selten, weniger als ein oder zwei Tage, Einige Tage, Mehr als sieben Tage, Fast jeden Tag in den letzten zwei Wochen

1. Ich habe mich schwindlig, benommen oder schwach gefühlt, wenn ich Nachrichten über das Coronavirus gelesen oder gehört habe.
2. Ich hatte Ein- oder Durchschlafstörungen, weil ich an das Coronavirus gedacht habe.
3. Ich habe mich gelähmt oder erstarrt gefühlt, wenn ich an das Coronavirus gedacht habe oder Informationen darüber ausgesetzt war.
4. Ich hatte keinen Appetit, wenn ich an das Coronavirus gedacht habe oder Informationen darüber ausgesetzt war.
5. Ich litt an Übelkeit oder Magen-Darm-Beschwerden, wenn ich an das Coronavirus gedacht habe oder Informationen darüber ausgesetzt war.

PAS

Antwortformat: Starke Ablehnung, Ablehnung, Weder Ablehnung noch Zustimmung, Zustimmung, Starke Zustimmung

1. Ich bin besorgt, dass ich mich mit Covid-19 infiziere.
2. Ich bin besorgt, dass meine Familie und Freunde sich mit Covid-19 infizieren.
3. Ich habe zurzeit Angst das Haus zu verlassen.
4. Ich bin besorgt, dass ich jemand anderen mit Covid-19 infizieren könnte.
5. Ich mache mir Sorgen, dass ich durch Covid-19 nicht arbeiten / nicht zur Schule gehen kann.
6. Ich mache mir Sorgen über mein derzeitiges Einkommen aufgrund von Covid-19.
7. Ich mache mir Sorgen über die langfristigen Auswirkungen von Covid-19 auf meine Berufsaussichten und die wirtschaftliche Situation.

CSS Subskala Danger

Instruktion: Im Folgenden werden verschiedene Arten von Sorgen abgefragt, die Sie in den letzten sieben Tagen möglicherweise hatten.
Antwortformat: Überhaupt nicht, Etwas, Mäßig, Sehr, Extrem

1. Ich bin besorgt, mich mit dem Coronavirus zu infizieren.

2. Ich bin besorgt, dass einfache Hygienemaßnahmen (z.B. Händewaschen) nicht ausreichen, um mich vor dem Coronavirus zu schützen.
3. Ich bin besorgt, dass unser Gesundheitssystem nicht in der Lage ist, mich vor dem Coronavirus zu schützen.
4. Ich bin besorgt, dass ich meine Familie nicht vor dem Coronavirus schützen kann.
5. Ich bin besorgt, dass unser Gesundheitssystem nicht in der Lage ist meine Angehörigen vor dem Coronavirus zu schützen.
6. Ich bin besorgt, dass soziale Distanzierung nicht ausreichend ist, um mich vor dem Coronavirus zu schützen.
