# Supplementary material for: Measuring COVID-19 Related Anxiety in Parents: Psychometric Comparison of Four Different Inventories
Source: JMIR Ment Health. 2020 Dec 3;7(12):e24507. doi: 10.2196/24507 (PMC7717922; doi:10.2196/24507)

Supplementary Materials

CAS-1 – ICC

CAS-2 – ICC

CAS-3 – ICC

CAS-4 – ICC

CAS-5 – ICC

CAS – Total Information Curve

CAS – Test Characteristic Curve

CSS-D1 – ICC

CSS-D2 – ICC

CSS-D3 – ICC

CSS-D4 – ICC

CSS-D5 – ICC

CSS-D6 – ICC

CSS-D – Total Information Curve

CSS-D – Test Characteristic Curve

FCV-19S-1 – ICC

FCV-19S-2 – ICC

FCV-19S-3 – ICC

FCV-19S-4 – ICC

FCV-19S-5 – ICC

FCV-19S-6 – ICC

FCV-19S-7 – ICC

FCV-19S – Total Information Curve

FCV-19S – Test Characteristic Curve

PAS-1 – ICC

PAS-2 – ICC

PAS-3 – ICC

PAS-4 – ICC

PAS – Total Information Curve

PAS – Test Characteristic Curve

CAS-1 (ICC)

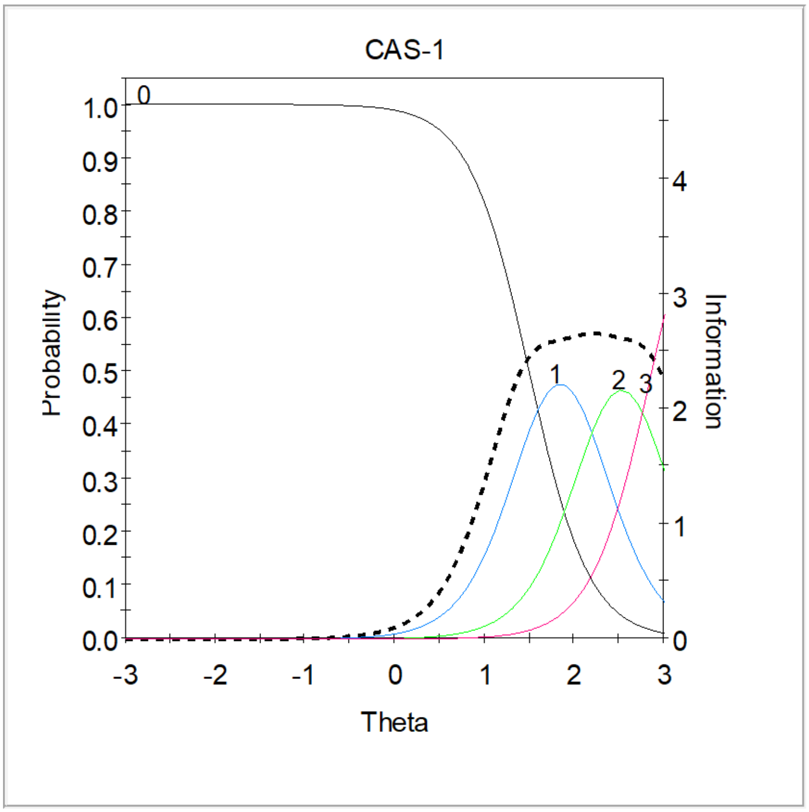

CAS-2 (ICC)

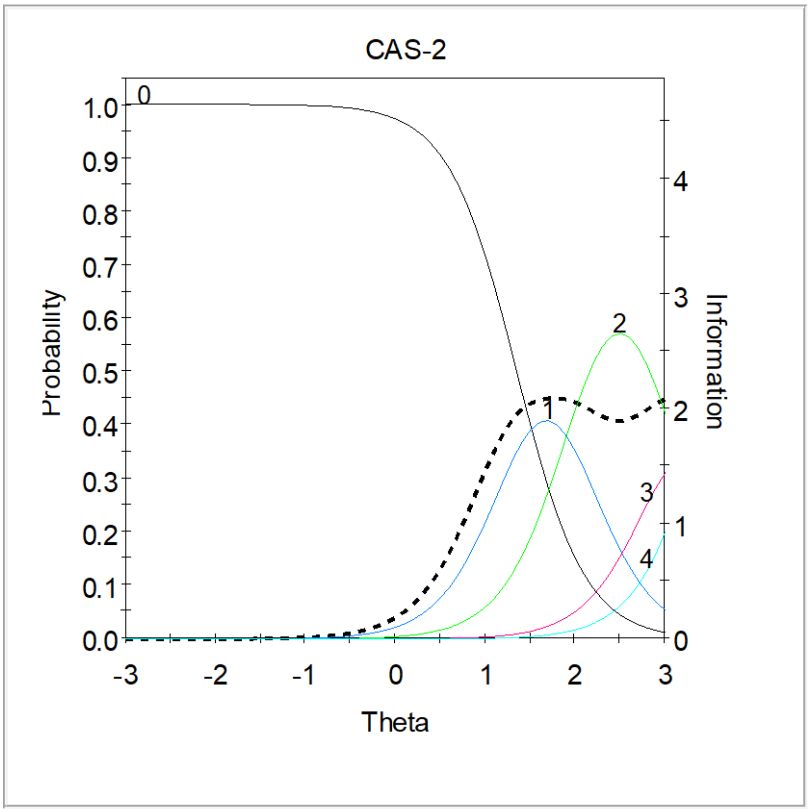

CAS-3 (ICC)

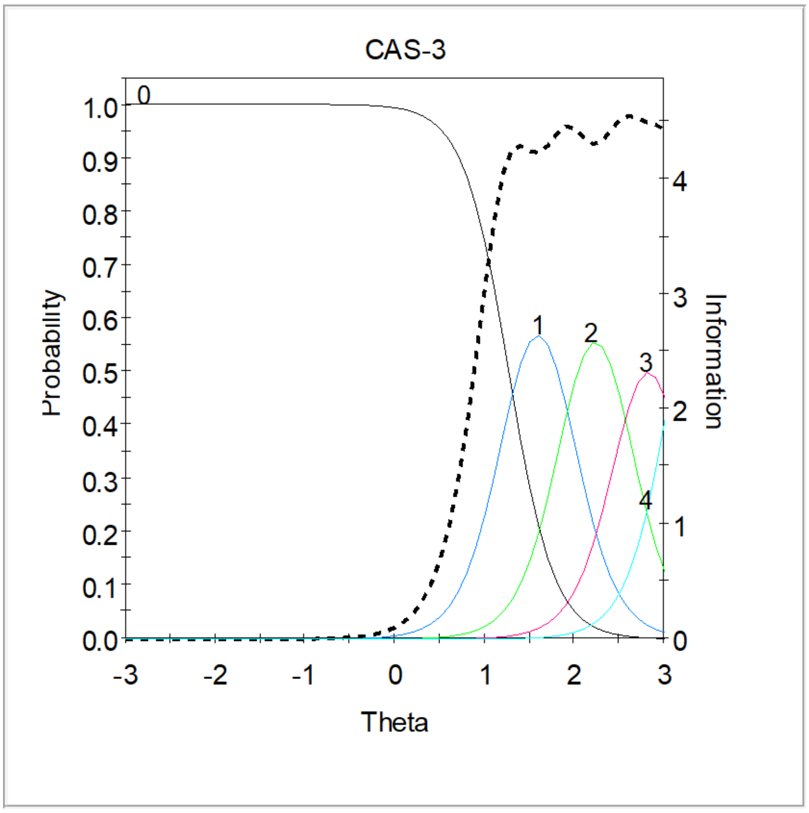

CAS-4 (ICC)

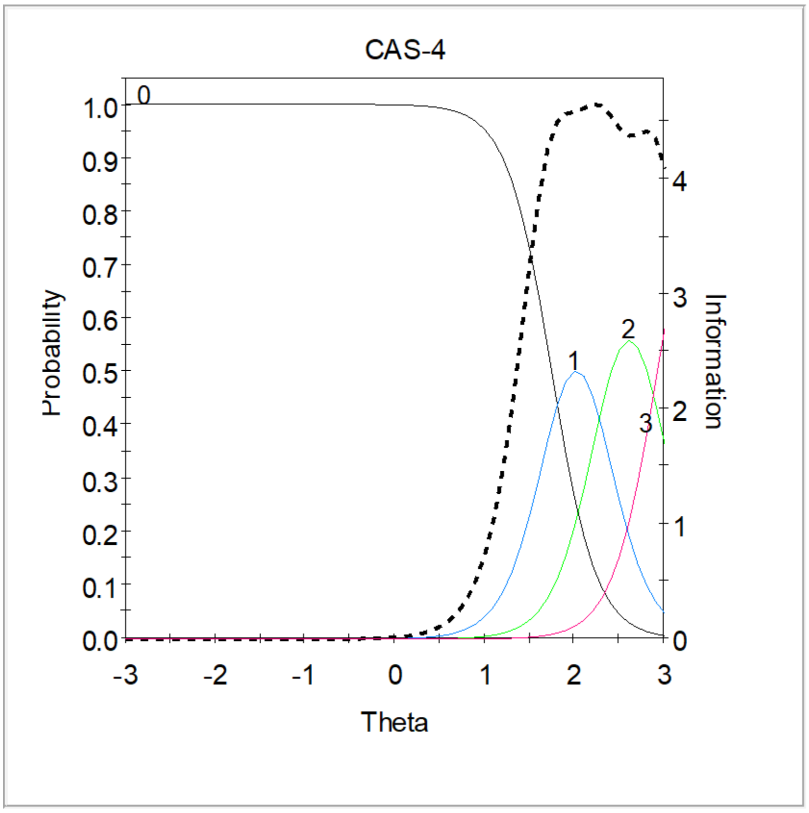

CAS-5 (ICC)

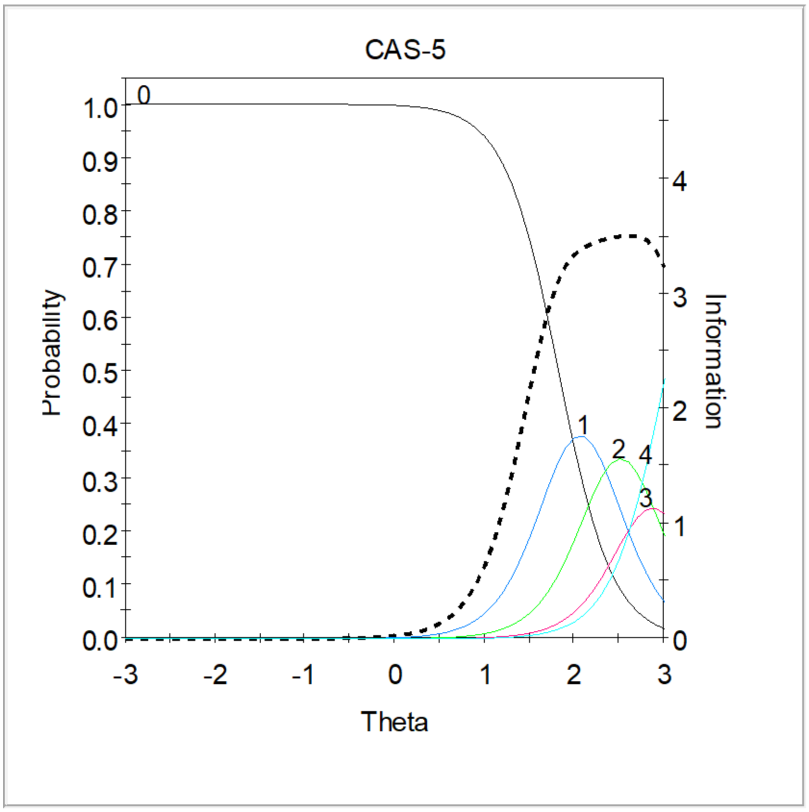

CAS – Total Information Curve

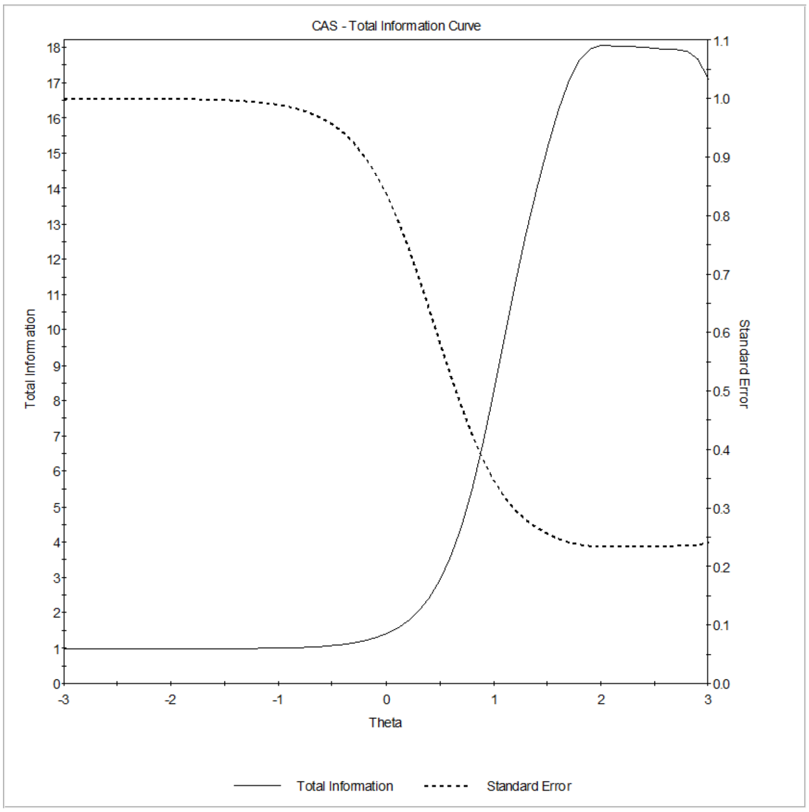

CAS – Test Characteristic Curve

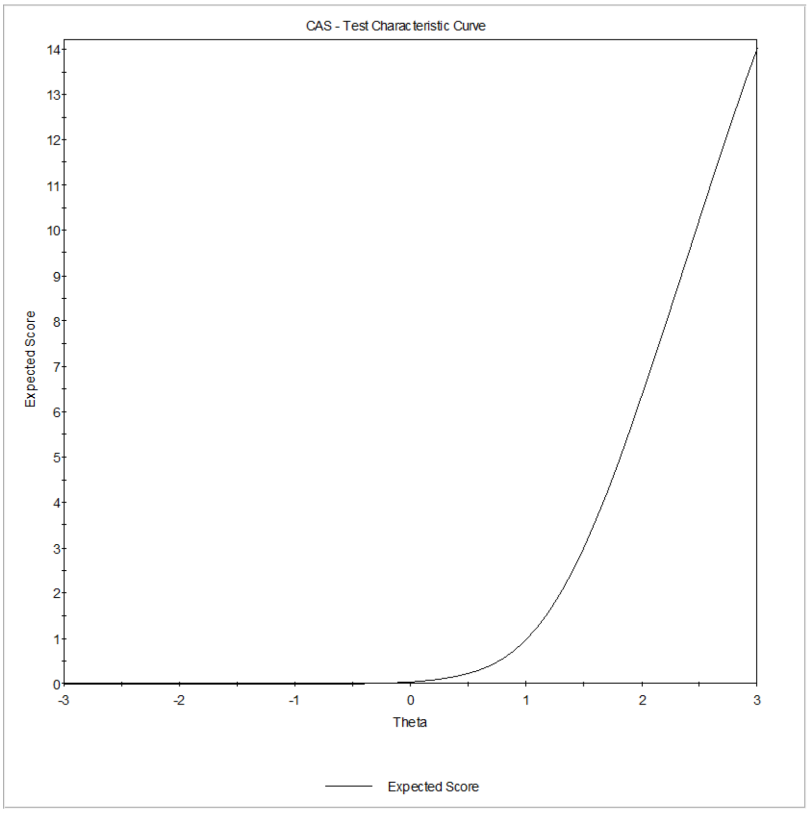

CSS-D1 (ICC)

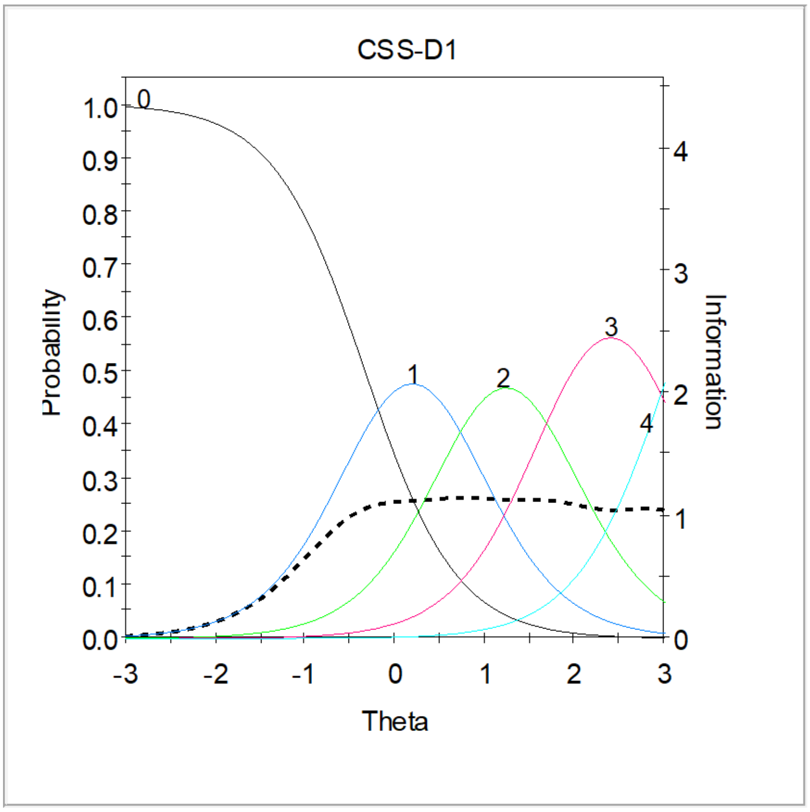

CSS-D2 (ICC)

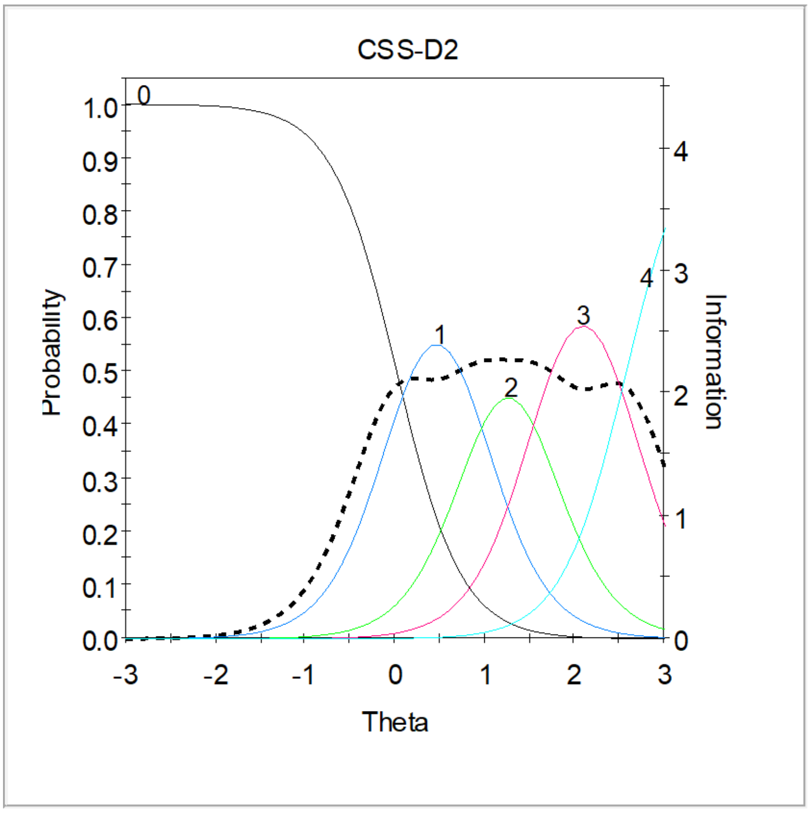

CSS-D3 (ICC)

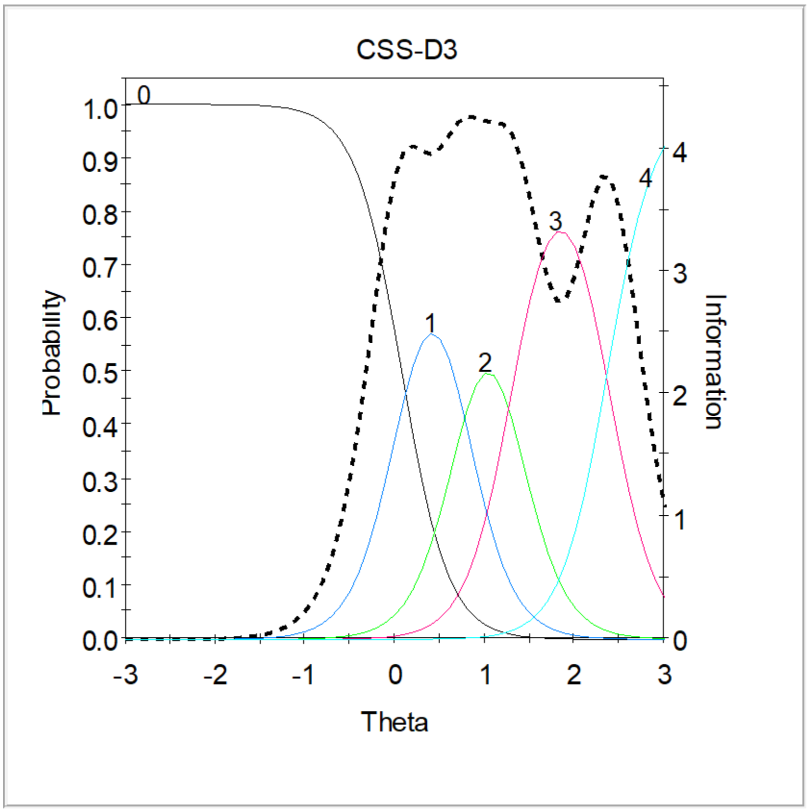

CSS-D4 (ICC)

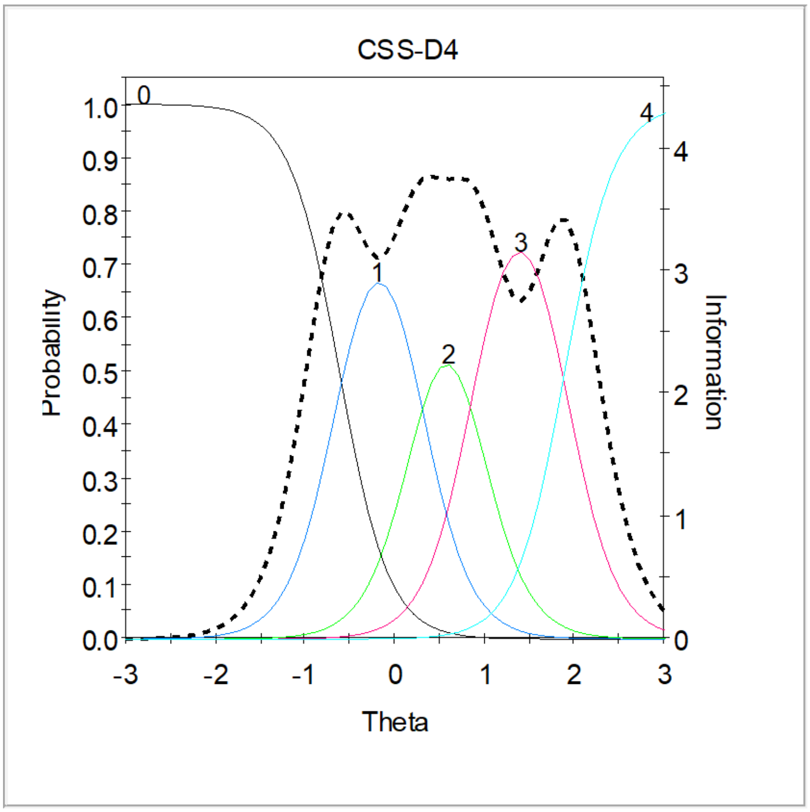

CSS-D5 (ICC)

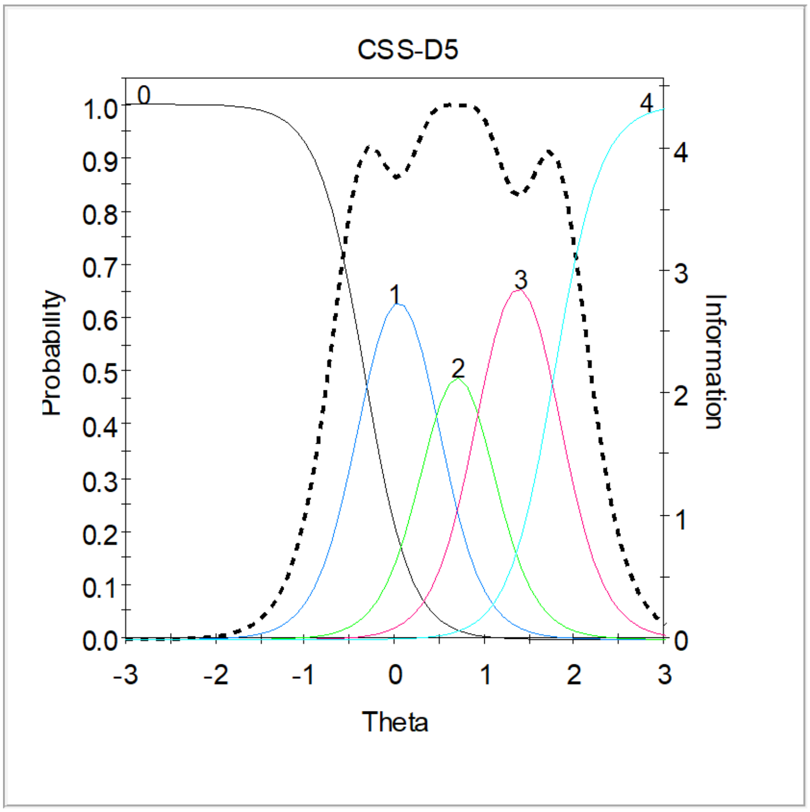

CSS-D6 (ICC)

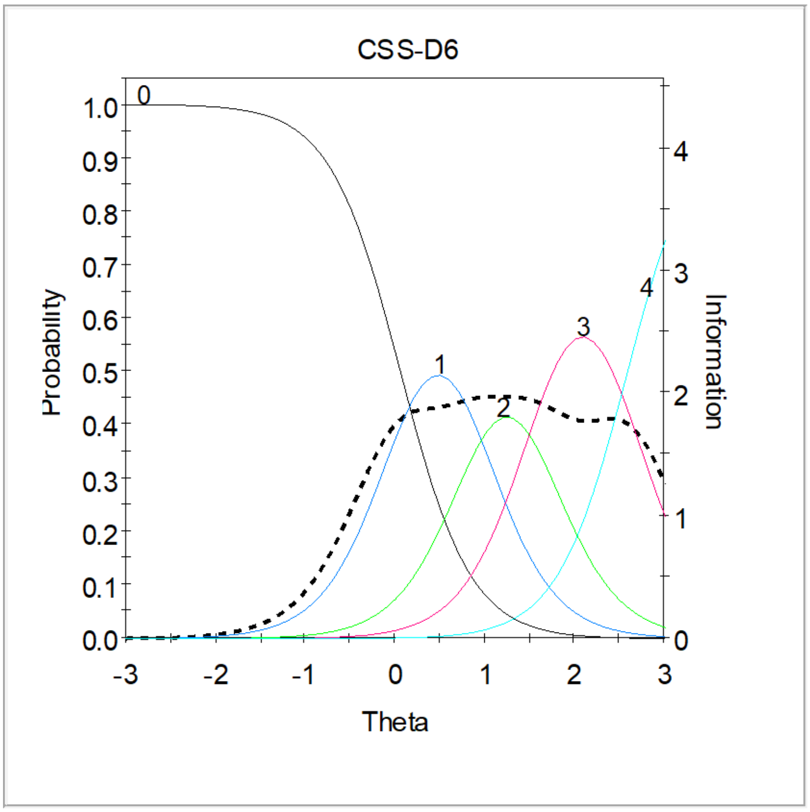

CSS-D – Total Information Curve

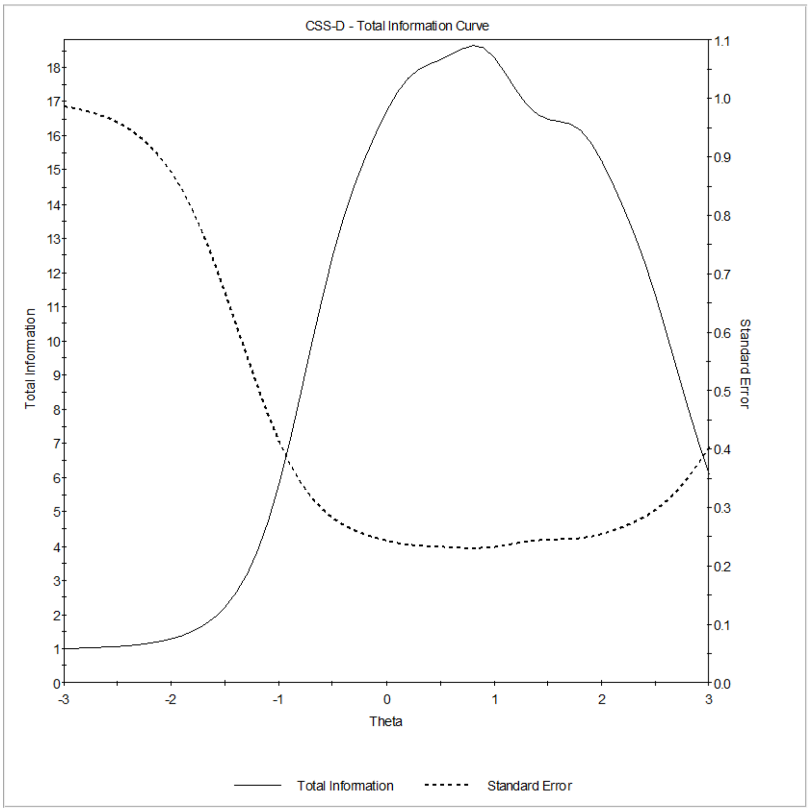

CSS-D – Test Characteristic Curve

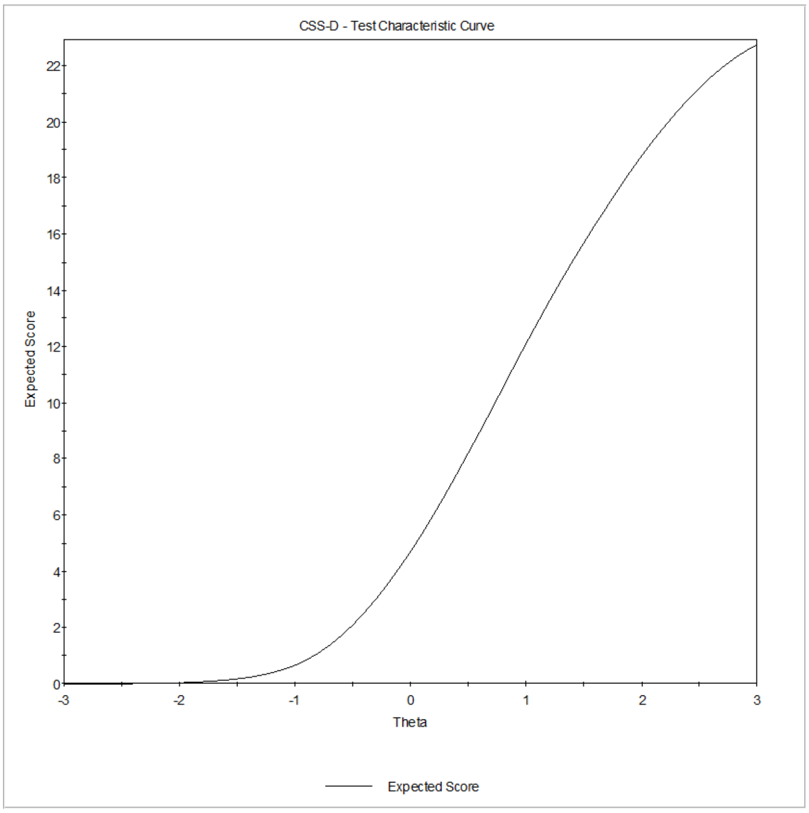

FCV-19S-1 (ICC)

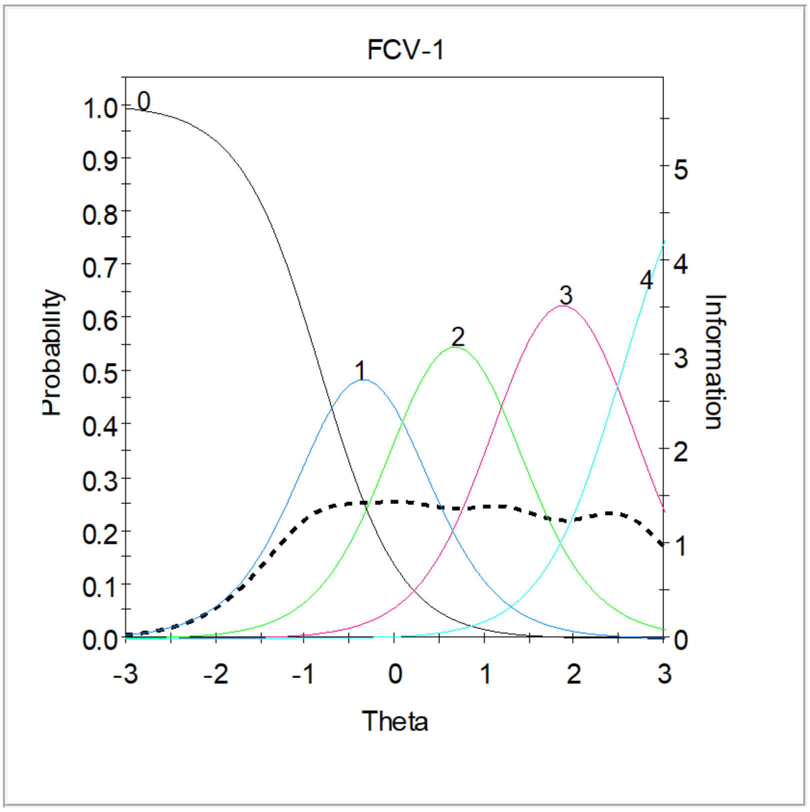

FCV-19S-2 (ICC)

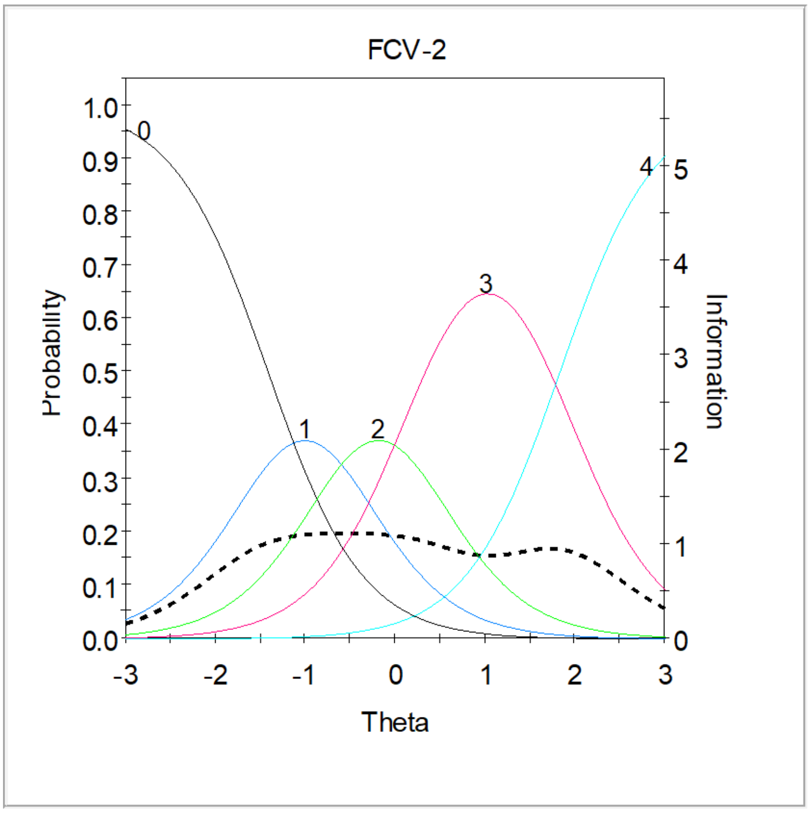

FCV-19S-3 (ICC)

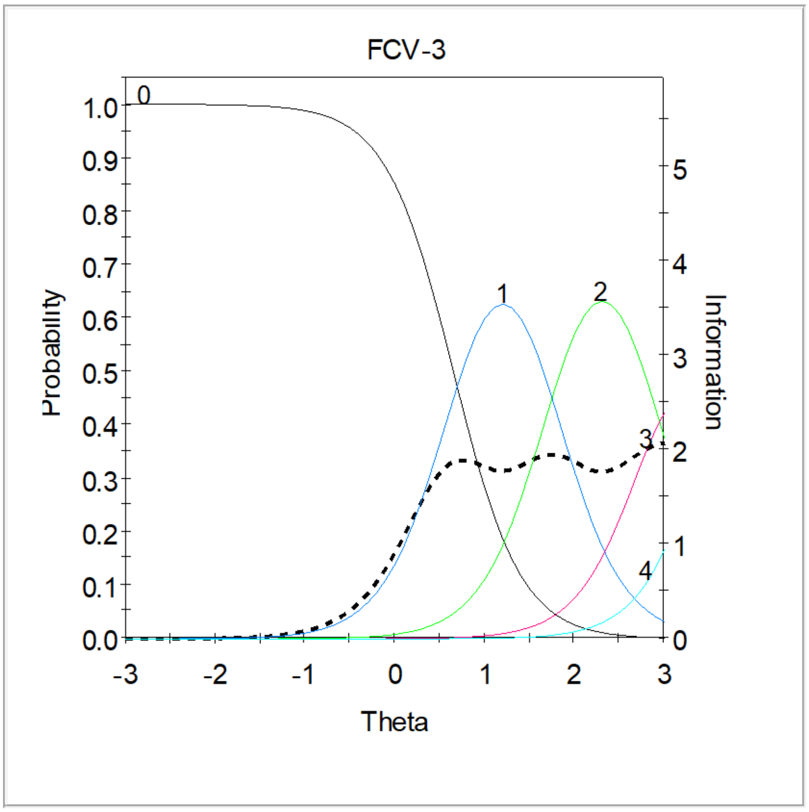

FCV-19S-4 (ICC)

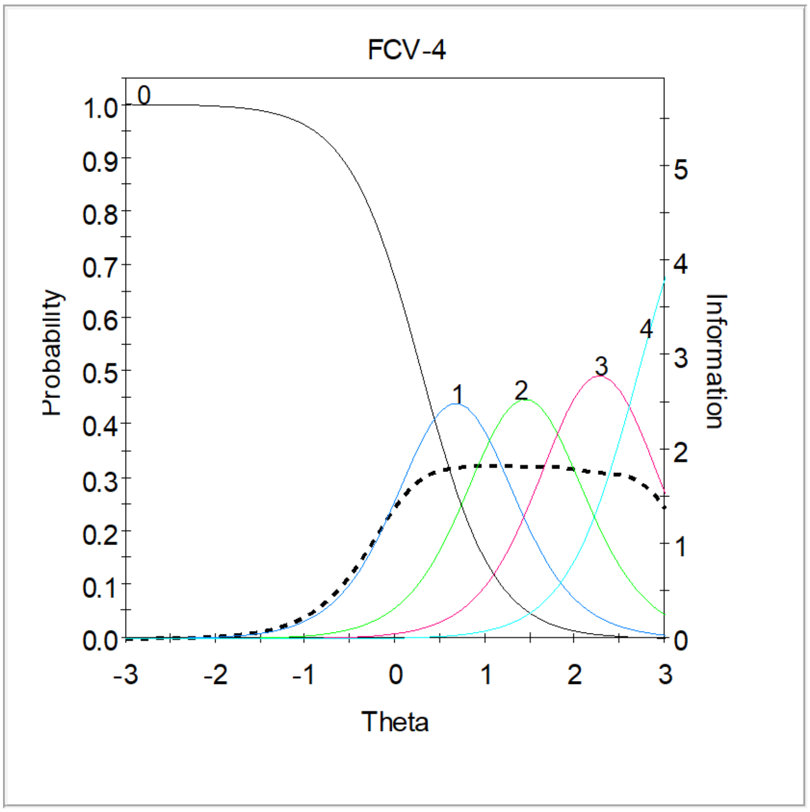

FCV-19S-5 (ICC)

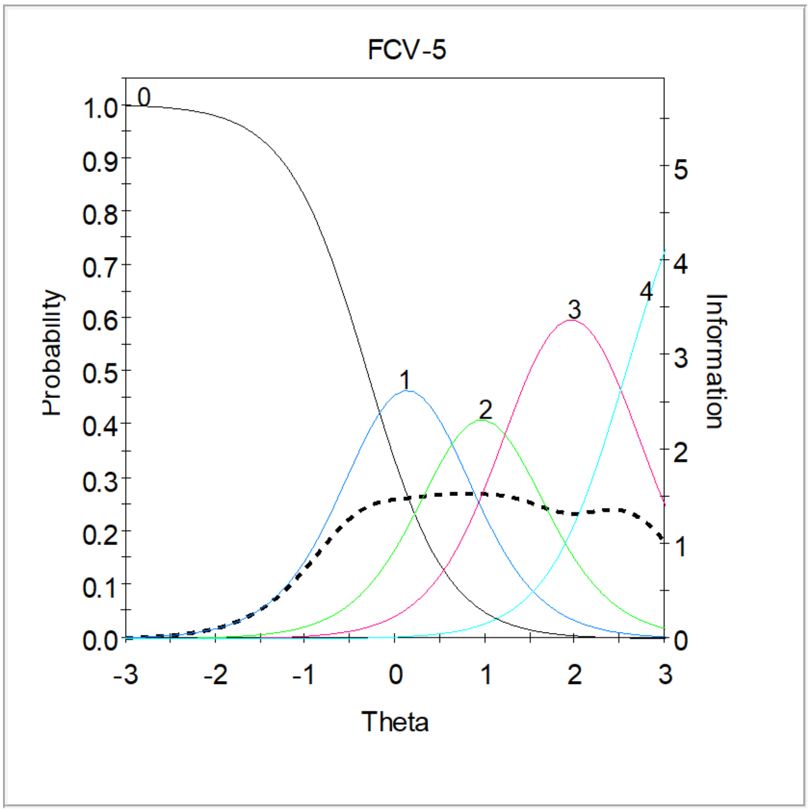

FCV-19S-6 (ICC)

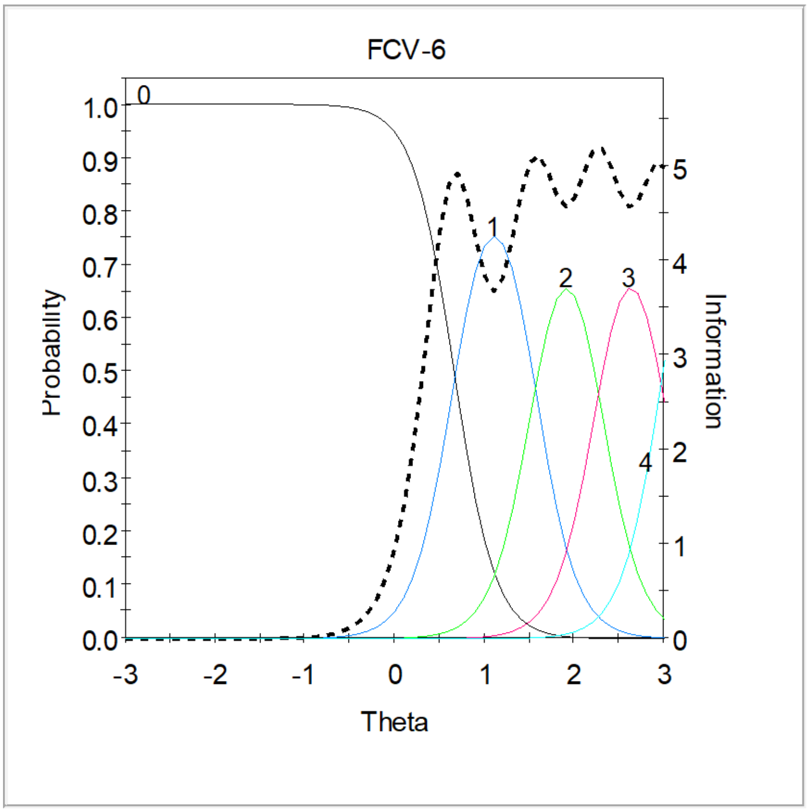

FCV-19S-7 (ICC)

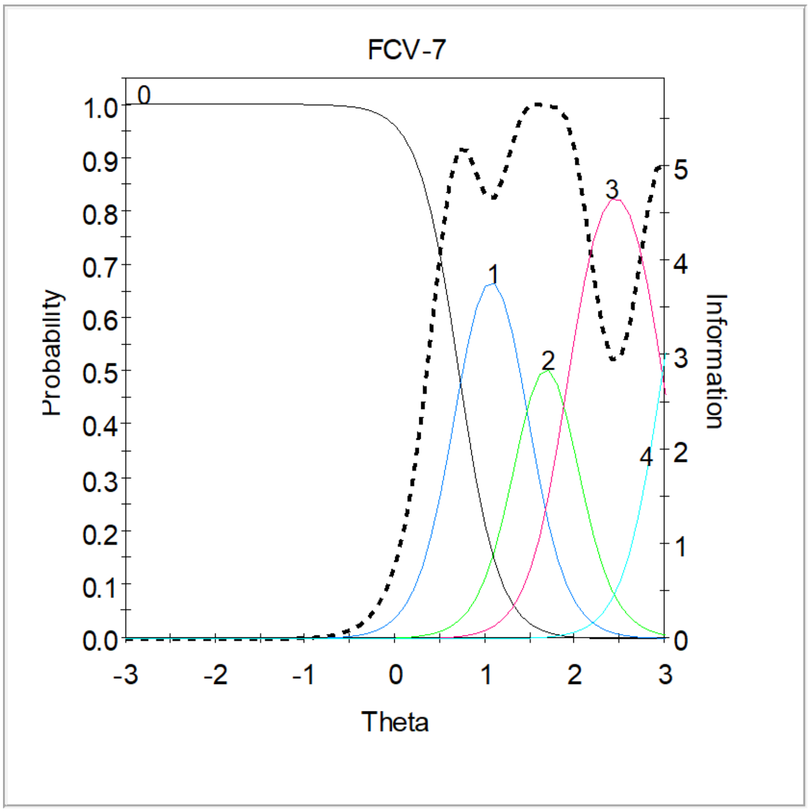

FCV-19S – Total Information Curve

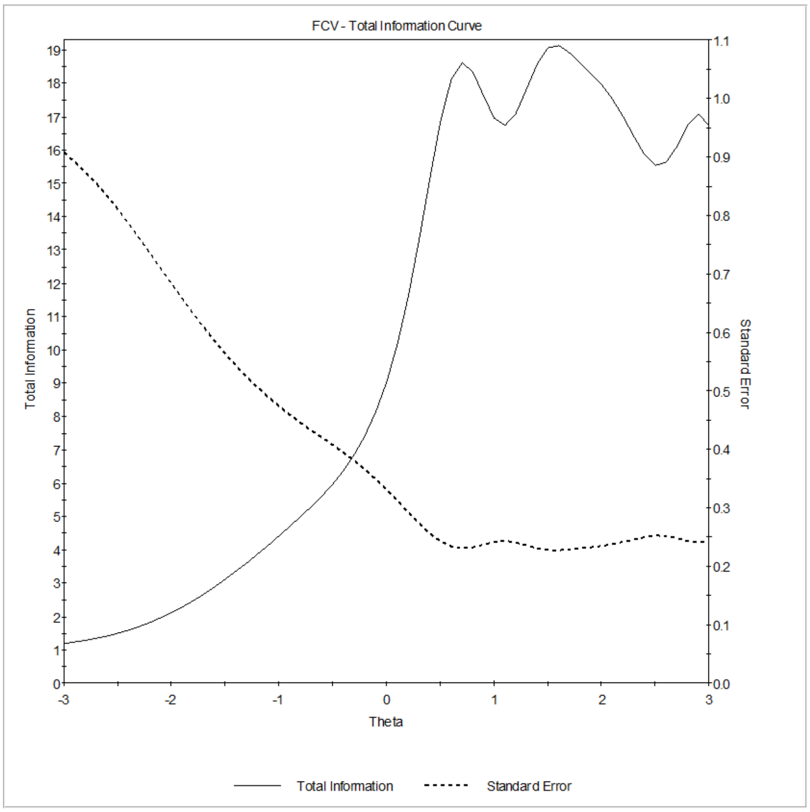

FCV-19S – Test Characteristic Curve

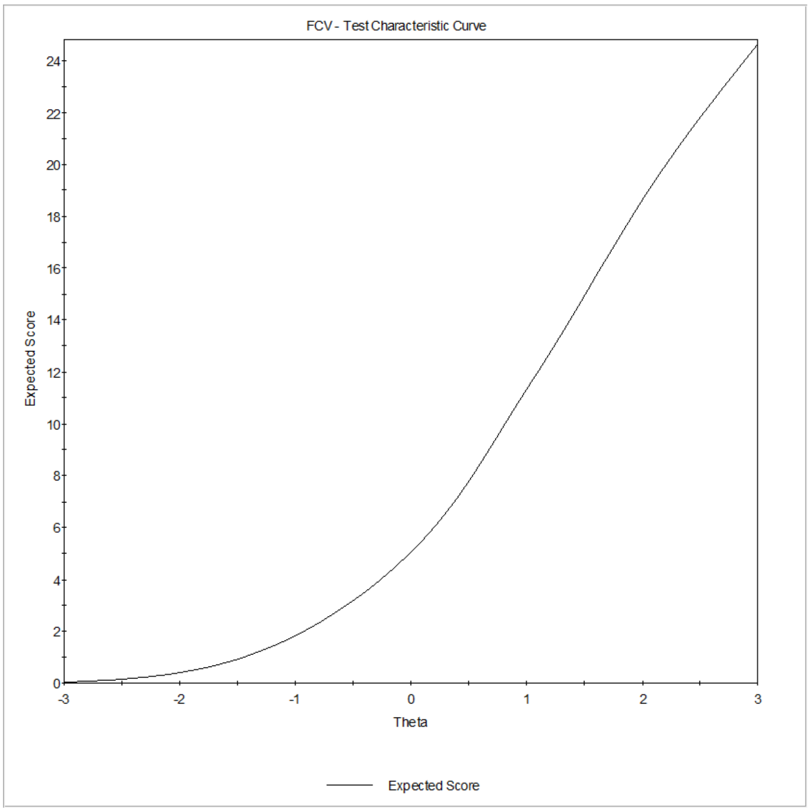

PAS-1 (ICC)

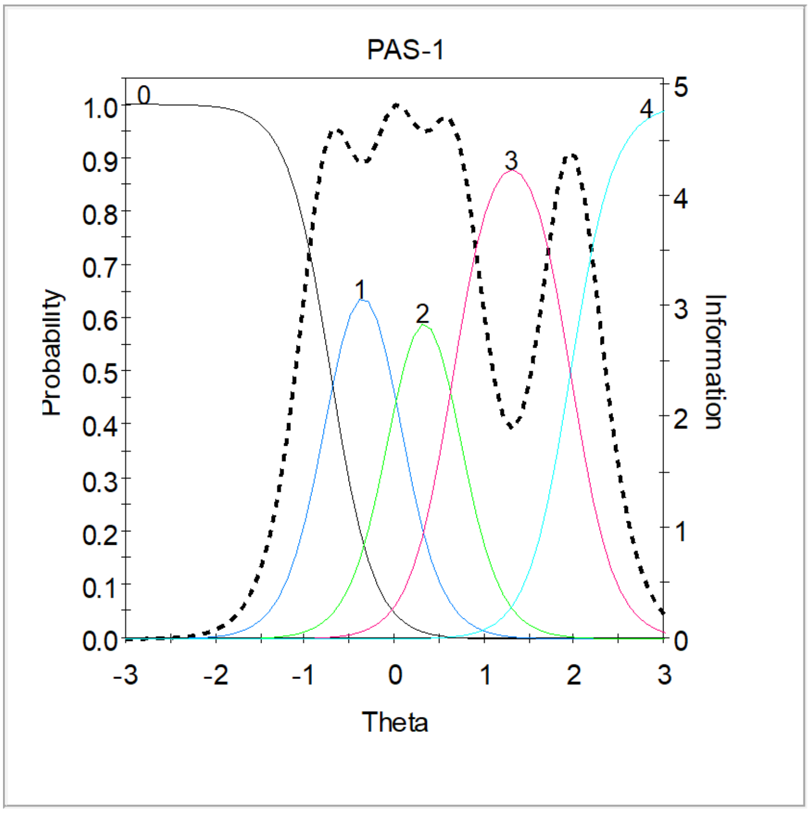

PAS-2 (ICC)

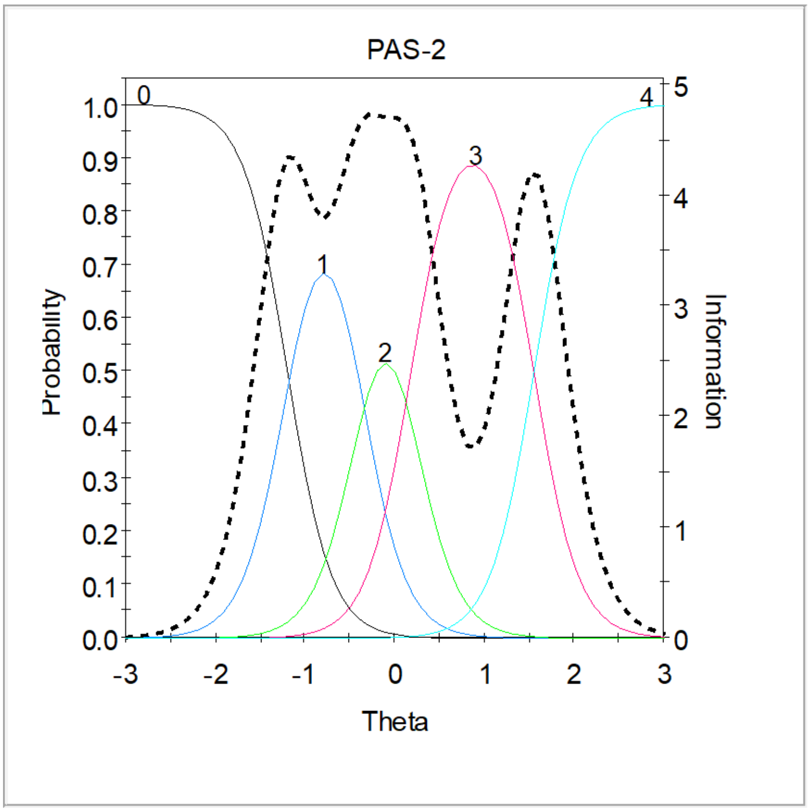

PAS-3 (ICC)

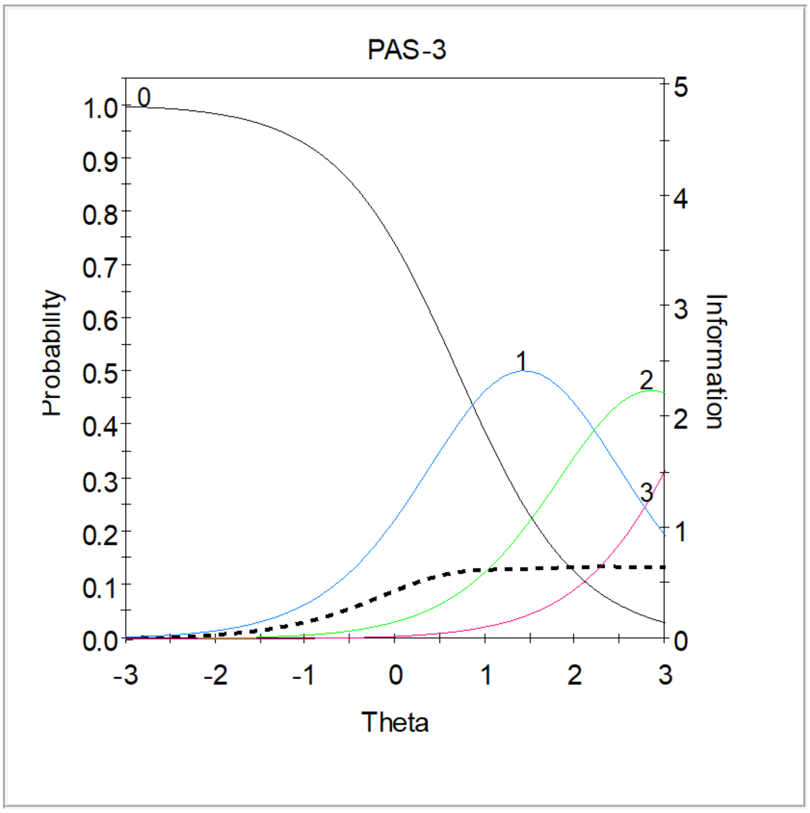

PAS-4 (ICC)

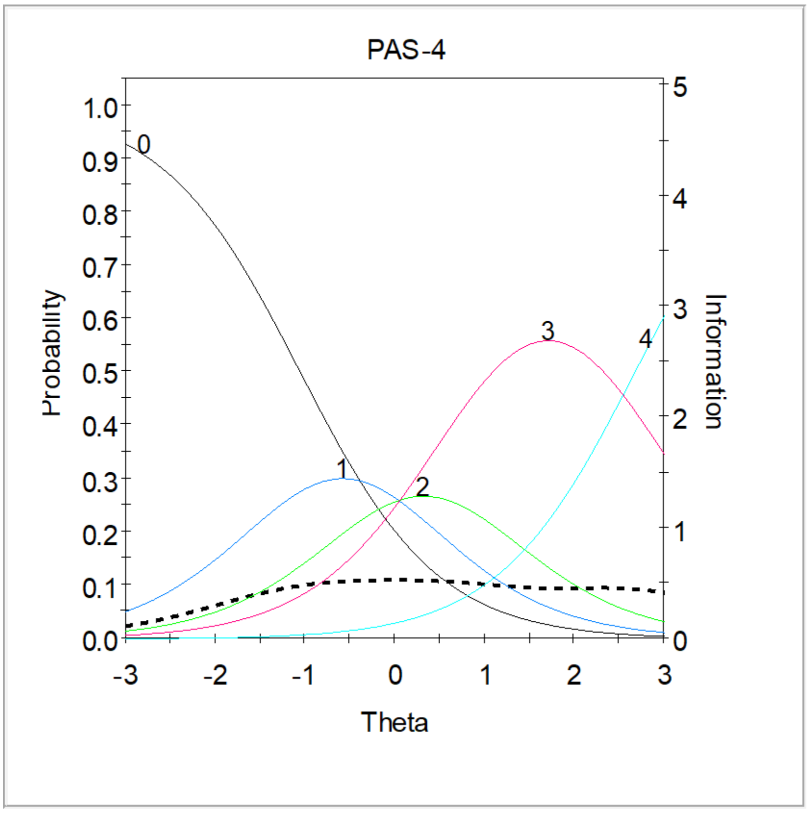

PAS – Total Information Curve

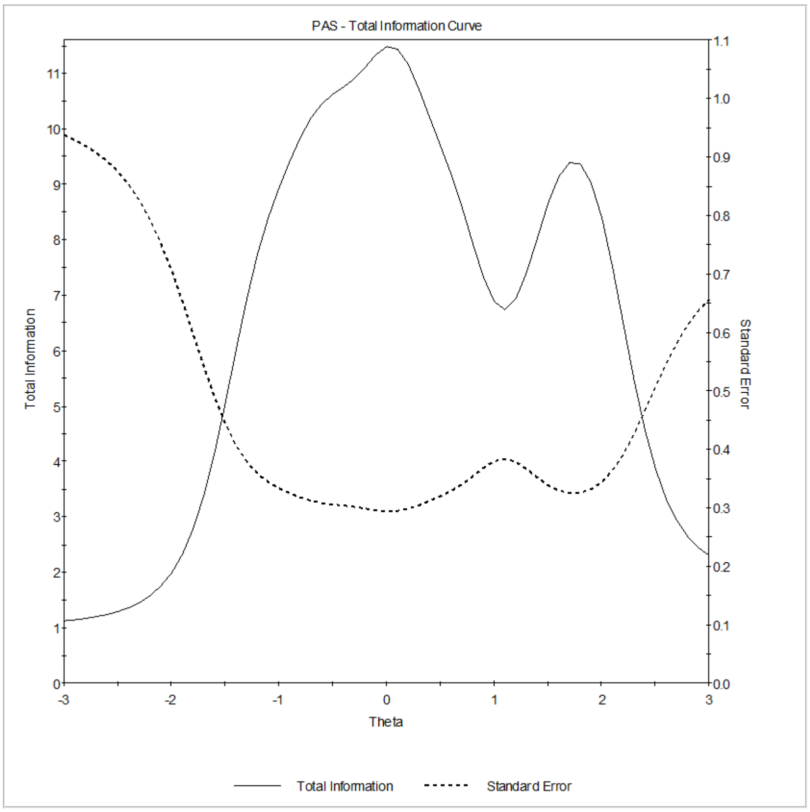

PAS – Test Characteristic Curve

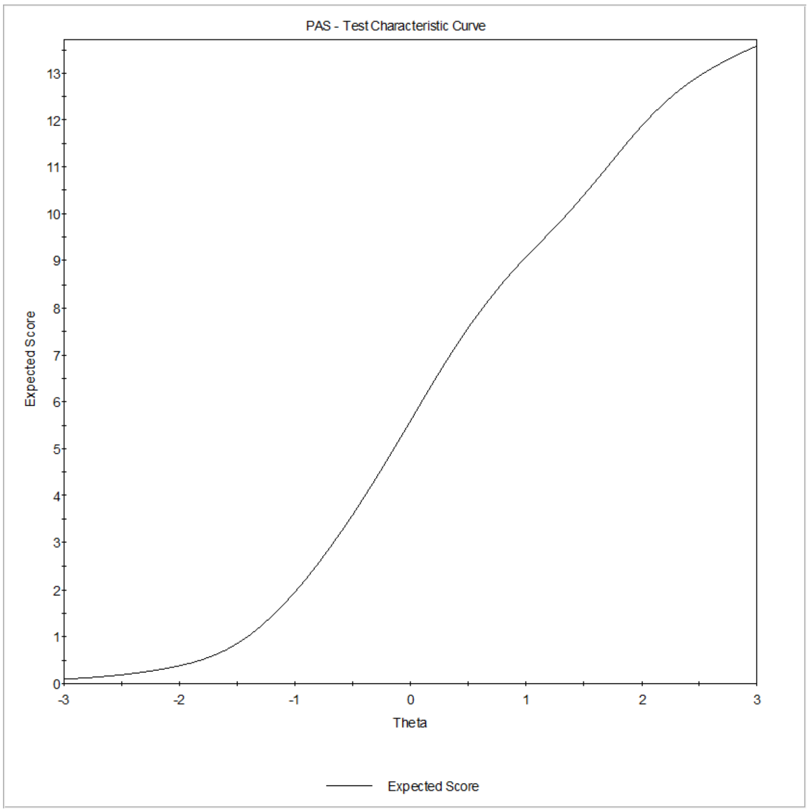

Supplement: Multimedia Appendix 2 [file mental_v7i12e24507_app2.pdf]
